# The role of glycoconjugates as receptors for insecticidal proteins

**DOI:** 10.1093/femsre/fuad026

**Published:** 2023-06-02

**Authors:** Hannah L Best, Lainey J Williamson, Emily A Heath, Helen Waller-Evans, Emyr Lloyd-Evans, Colin Berry

**Affiliations:** School of Biosciences, Cardiff University, Museum Avenue, Cardiff CF10 3AX, United Kingdom; School of Biosciences, Cardiff University, Museum Avenue, Cardiff CF10 3AX, United Kingdom; School of Biosciences, Cardiff University, Museum Avenue, Cardiff CF10 3AX, United Kingdom; Medicines Discovery Institute, Cardiff University, Park Place, Cardiff CF10 3AT, United Kingdom; School of Biosciences, Cardiff University, Museum Avenue, Cardiff CF10 3AX, United Kingdom; School of Biosciences, Cardiff University, Museum Avenue, Cardiff CF10 3AX, United Kingdom

**Keywords:** *Bacillus thuringiensis*, insecticidal, pesticidal, glycobiology, toxins

## Abstract

*Bacillus thuringiensis* (Bt) proteins are an environmentally safe and effective alternative to chemical pesticides and have been used as biopesticides, with great commercial success, for over 50 years. Global agricultural production is predicted to require a 70% increase until 2050 to provide for an increasing population. In addition to agriculture, Bt proteins are utilized to control human vectors of disease—namely mosquitoes—which account for >700 000 deaths annually. The evolution of resistance to Bt pesticial toxins threatens the progression of sustainable agriculture. Whilst Bt protein toxins are heavily utilized, the exact mechanisms behind receptor binding and toxicity are unknown. It is critical to gain a better understanding of these mechanisms in order to engineer novel toxin variants and to predict, and prevent, future resistance evolution. This review focuses on the role of carbohydrate binding in the toxicity of the most utilized group of Bt pesticidal proteins—three domain Cry (3D-Cry) toxins.

## Introduction

*Bacillus thuringiensis* (Bt) is a Gram-positive bacterium that produces a large variety of insecticidal δ-endotoxins during sporulation. These proteins may be lethal to insects and/or nematodes yet are innocuous to vertebrates and plants. Additionally, Bt proteins demonstrate species-specific activity, allowing for the eradication of harmful pests that destroy crops and spread disease without exterminating beneficial insect species. Bt proteins are an environmentally safe and effective alternative to chemical pesticides and have now been used as biopesticides for over 50 years. In addition, genes encoding Bt proteins have been incorporated in crops such as corn and cotton with huge commercial success (Sandhu et al. 2020). The exact mechanisms behind Bt protein(s) toxicity are unknown, and increasing understanding is critical for the development of new Bt proteins, and to counteract emerging field resistance.

Bt pesticidal proteins may be produced during sporulation (crystal and cytolytic proteins) or the vegetative growth phase and are generally organized into a number of categories based on structural families, according to a recently revised nomenclature (Crickmore et al. 2021). The 3D-Cry toxins form the largest known group and are also the most mechanistically well-characterized—especially those that are lepidopteran active. Following ingestion by invertebrates, 3D-Cry activity is proposed to occur by either of two models; the most-widely known sequential binding pore-forming (Schnepf and Whiteley 1981, Bravo et al. 2007, Rodriguez-Almazan et al. 2009) or the alternative G-protein mediated apoptotic signalling pathway model (Zhang et al. 2006, Castella et al. 2019, Mendoza-Almanza et al. 2020). In the sequential binding model, Cry crystals are solubilized in the specific pH and physiological conditions of the insect gut, producing monomeric protoxins. The monomers are subsequently activated by host proteinases, yielding activated Cry proteins, which bind target receptors on the brush border membranes of midgut epithelial cells. This is followed by cleavage within the α-helical domain I by host proteinases, triggering toxin oligomerization to form a prepore structure necessary for insertion into the phospholipid bilayer to form a channel. This culminates in cell death via colloid-osmotic lysis. There is increasing evidence that other routes to pore formation via receptor binding may exist and that the sequential binding model may not be a universal pathway (Vachon et al. 2012, Endo et al. 2022, Sun et al. 2022). The signalling model differs in that there is no pore insertion, with cell death induced, instead, via the activation of an apoptotic signalling cascade—although this is not a widely accepted hypothesis.

Although significantly different at the amino acid level, active 3D-Cry proteins have a characteristic conserved 3-domain architecture (D-I–D-III) indicative of a similar mechanism of action. Crystal structures are available for a number of activated 3D-Cry (Cry1Aa (Grochulski et al. 1995), Cry1Ac (Derbyshire et al. 2001), Cry2Aa (Morse et al. 2001), Cry3Aa (Heater et al. 2020), Cry3Bb1(Galitsky et al. 2001), Cry4Aa (Boonserm et al. 2006), Cry4Ba (Boonserm et al. 2005), Cry5Ba (Hui et al. 2012), Cry7Ca1 (Jing et al. 2019), and Cry8Ea1 (Guo et al. 2009) along with a number of mutant and chimeric forms) and all show a conserved structural arrangement. Domain I is linked to pore formation and consists of a helical bundle with a central hydrophobic helix-α5, associated with initializing membrane insertion, encapsulated by six amphipathic helices. Domains II and III are associated with receptor binding and are β-sheet-rich domains resembling lectins. Both domains present structural homology to carbohydrate binding proteins, such as lectin jacalin and sialidase, respectively. This structural similarity implies that carbohydrate residues may play a critical role in receptor binding for 3D-Cry proteins—although the exact mechanisms by which this occurs remain somewhat unknown. The 3D crystal structure of the Cry1Ac1 protoxin has recently been elucidated, presenting four cysteine-rich prodomains (D-IV–D-VII) (Evdokimov et al. 2014). Domains IV and VI are alpha helical bundles that resemble spectrin or bacterial fibrinogen-binding complement inhibitor, whilst D-V and D-VII are beta-rolls that closely resemble the carbohydrate-binding moieties seen in sugar hydrolases of Family 6 carbohydrate binding module—and similar to that seen in D-II and D-III. Aside from a few recent investigations (Zghal et al. 2017, Pena-Cardena et al. 2018), prodomain studies have largely indicated that it is dispensable for insecticidal activity, and instead has roles in optimizing crystal formation, packing different toxin variants into the same crystal, stability, selective solubilization, and ensuring synchronous delivery through oligomerization (Luthy and Ebersold 1981, Hofte and Whiteley 1989, Evdokimov et al. 2014).

Cry proteins are usually highly selective to their target insect orders, and it is unusual to find a Cry protein that effectively targets more than one order—although exceptions exist, such as Cry2Aa, which has activity against Lepidoptera (Donovan et al. 1988) and Diptera (Yamamoto and Mclaughlin 1981b), and Cry1Ba which has been shown to target Hemiptera (Fernandez-Luna et al. 2019), Lepidoptera (Simpson et al. 1997), and Diptera and Coleoptera (Zhong et al. 2000). As well as the unique domain structure in individual Cry proteins, target selectivity is determined by the presence of the receptor proteins and lipids in the target insect midgut. A relatively strong understanding of this process has been derived in Lepidoptera, where several protein types have been identified to function as Cry receptors, including; cadherin-like proteins (CAD; Nagamatsu et al. 1998, Vadlamudi et al. 1993, 1995, Gahan et al. 2001), GPI-anchored aminopeptidases (APN; Sangadala et al. 1994, Gill et al. 1995, Rajagopal et al. 2002, Knight et al. 2004), GPI-anchored alkaline phosphatases (ALP; Sangadala et al. 1994, Jurat-Fuentes and Adang 2004), and ABC transporters (Sato et al. 2019). Similar receptors have been identified in other orders, e.g. mosquitoes (Diptera) utilize cadherins (Cry4Ba, Cry11Ba, and Cry11Aa), APNs (Cry11Ba), and ALPs (Cry11Aa). A series of more recent work has identified that glycosphingolipids (GSLs) can also function as Cry5B and Cry14A receptors and mediate toxicity in the nematode *Caenorhabditis elegans* (Griffitts et al. 2003, 2005).

Resistance development against insecticidal toxins is a common phenomenon, and a wide array of resistance mechanisms has been identified from both laboratory and field studies (Peterson et al. 2017). The most common mechanism appears to be altered Cry binding to receptors (Ferre and Van Rie 2002). Cadherins have received substantial attention due to their commonality as lepidopteran receptors and major mutations causing significant resistance to Cry1Ac have been identified in multiple strains of *Heliothis virescens* (Gahan et al. 2001), *Pectinophora gossypiella* (Morin et al. 2003, Tabashnik et al. 2004, 2005, Fabrick and Tabashnik 2012, Fabrick et al. 2014), and *Helicoverpa armigera* (Xu et al. 2005, Yang et al. 2006, Zhao et al. 2010, Zhang et al. 2013), yet it is clear that cadherin binding and expression can be identical between resistant and susceptible strains (Siqueira et al. 2006, Bel et al. 2009). This, alongside other studies, has led to the common hypothesis that a combination of other putative Cry binding moieties, such as APNs, ALPs, GSLs, and so on, may be required for full toxicity.

This review will focus on appraisal of the literature surrounding the relevance of carbohydrate moieties in eliciting the insecticidal action of 3D-Cry proteins. In addition to the aforementioned Cry5B and Cry14A, there is ample precedent for the role of glycoconjugates as receptors for protein toxins—as is the case for cholera toxin (Holmgren et al. 1975, Kabbani et al. 2020), aerolysin (Abrami et al. 2002), shiga toxin (Smith et al. 2006), and ricin (Sandvig et al. 1976). To understand how Cry toxins exploit carbohydrate moieties for toxicity in more detail, we will also provide a beginner’s overview to the current understanding of the structural diversity, biosynthesis, and function of insect glycoconjugates, as well as comparing insect glycopatterning to the better-characterized pathways and glycoconjugate species present in mammals.

## Glycoprotein glycans in insects and nematodes

The addition of an oligosaccharide chain to a protein backbone (glycosylation) is an extremely common posttranslational modification in eukaryotes. A substantial array of studies have concluded that glycoprotein moieties play critical roles in cell signalling, cell migration, cell–cell interactions, blood group determination, and immune cell trafficking—with changes in N-glycosylation associated with diverse disorders including cancers (Kodar et al. 2012), Crohn’s disease (Verhelst et al. 2020), and diabetic kidney disease (Bermingham et al. 2018). The distinct and divergent glycosylation patterns observed are driven by an orchestra of glycosidases and glycosyltransferases, which differ in terms of substrate specificity, and both temporal and spatial expression. The exact size and structure of the oligosaccharide can dramatically alter the biophysical properties of the protein—effectively significantly diversifying the functions of a single gene product.

As with vertebrates, insects and nematodes demonstrate both major forms of glycosylation; N-linked (attached to Asn in an Asn-X-Ser motif, where X is not Pro) and O-linked (attached via Ser/Thr). As in mammals, insect and nematode N-linked glycosylation begins in the endoplasmic reticulum (ER) with the cotranslational transfer of a dolichol-linked precursor oligosaccharide to the asparagine side chain of the consensus sequence within a nascent protein. This precursor is subsequently processed in multiple stages to form mature variants in the ER and Golgi. O-glycosylation also occurs in the ER, Golgi and, occasionally, the cytoplasm but unlike N-linked does not begin with a common oligosaccharide precursor.

The vast majority of knowledge on insect glycoconjugates comes from the model organism *Drosophila melanogaster* (order Diptera), although there are now, collectively, a generous number of studies on the glycomes of species within the orders Lepidoptera (Stanton et al. 2017, Cabrera et al. 2016, Fuzita et al. 2020), Hemiptera (Scheys et al. 2019), Hymenoptera (Hykollari et al. 2019), and Nematoda (Cipollo et al. 2005, Paschinger et al. 2008, Vanbeselaere et al. 2018, Wang et al. 2021). Genome completion of *Drosophila* and random mutagenesis studies have enabled the elucidation of putative genes for glycoconjugate biosynthesis and the functional impact of altering glycan patterning (Seppo and Tiemeyer 2000, Ten Hagen et al. 2009).

## N-linked protein glycosylation

**Figure 1.**
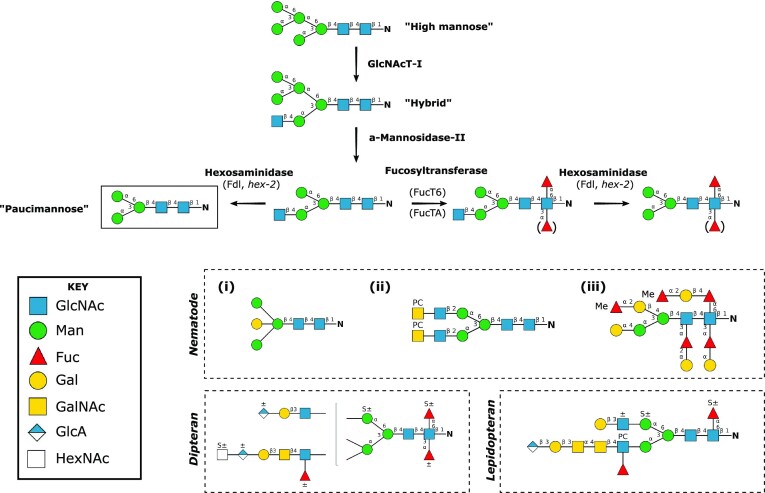
Overview of N-glycosylation in insects and nematodes. In insects and nematodes, high-mannose oligosaccharides are attached in the ER to consensus Asn residues and are subsequently processed by glycosidases and glycosyltransferases to generate a variety of N-linked structures. The synthetic pathway shown begins with the ER glycosidase-processed high mannose glycan (Man_5_GlcNAc_2_). N-linked diversity is limited through the expression of a hexosaminidase (Fdl in *Drosophila, hex-2 C. elegans*) generating paucimannose—one of the most predominant N-linked glycans in all characterized insecta. A common feature of insecta N-glycans—core fucosylation, occurs at C3 and/or C6 of the reducing terminal GlcNAc (via FucT6 and FucTA in *Drosophila*). N-linked diversity is expanded through the expression of less well-defined glycosyltransferases (and potentially sialyltransferases). Significant diversity and unique glycan signatures have been noted in complex N-glycans between different species. For example, nematode N-glycans can contain structures (i) with a bisecting galactose, (ii) with multiple phosphocholine (PC) residues as antennal modifications, and (iii) that are fucose rich with O-methylation (Me) modifications and the extension of core fucosylated residues. Nematode structures are based on figures from Paschinger et al. (2008), Haslam et al. (2002), and Wilson and Paschinger (2016). Dipteran N-glycans with example antennae modifications (as found in *Aedes aegypti, Anopheles gambiae*, and *D. melanogaster*; Kurz 2015, Paschinger and Wilson 2020) and an *L. dispar* zwitterionic lepidopteran N-glycan (Paschinger and Wilson 2020, Stanton 2017) show features frequently found in many insect species: sulphated residues (S±) and glucuronic acid attached to a galactose residue. Glycans are depicted according to the Symbolic Nomenclature for Glycans, as shown in **KEY**, linkages are shown next to the bonds, and known enzymes are named next to initial N-glycan trimming stages.

All N-glycans share the same pentasaccharide core, termed paucimannose (Man_3_GlcNAc_2_),—a core conserved from protozoan to metazoan. After the dolichol-linked precursor oligosaccharide (Glc_3_Man_9_GlcNAc_2_) has been transferred to the protein, resident ER glucosidases and mannosidase remove three glucose residues and a mannose residue, respectively. For most glycoproteins, mannose residues are further trimmed in the Golgi generating a high mannose structure (Man_5_GlcNAc_2_), followed by GlcNAc transferase (GlcNAcT-1)-mediated conversion into a hybrid glycan (GlcNAcMan_5_GlcNAc_2_), and mannosidase II-mediated conversion into GlcNAcMan_3_GlcNAc_2._ In invertebrates, this glycan can be trimmed further to generate paucimannose (Man_3_GlcNAc_2_; Fig. 1)—an N-glycan that has only rarely, and relatively recently, been detected in vertebrates (Lattova et al. 2010, Balog et al. 2012, Zipser et al. 2012). These initial trimming stages can be followed by additional enzymatic steps to add diverse sugar residues and generate more complex N-glycans.

Initial studies on N-linked glycans in *Drosophila* larvae and cultured *Drosophila* S2 cells showed a predominance of high (Man_5_GlcNAc_2_) and paucimannose (Man_3_GlcNAc_2_) moieties, suggesting an absence of more complex glycans (Parker et al. 1991, Williams et al. 1991). These simple N-glycans can be fucosylated via α1–6 and α1–3 linkages to the reducing terminal N-GlcNac. This is divergent from vertebrates where, although N-glycans have paucimannose as a core, the simplest N-glycan is chiefly GlcNAcMan_3_GlcNAc_2._ Furthermore, vertebrates only fucosylate N-glycans at the α1–6 linkage. Later work, after completion of the *Drosophila* genome, elucidated candidate glycosyltransferases required for the generation of more complex glycans. This, combined with improved analytical techniques, led to several mass spectrometry-based studies, which established the presence of hybrid, biantennary, and triantennary *Drosophila* glycoproteins—including sulphated, glucuronylated, and sialylated structures (Koles et al. 2004, North et al. 2006, Aoki et al. 2007)—although the degree of sialyation is hotly debated (Ghosh et al. 2018, Marchal et al. 2001), with the only published studies reporting N-linked sialylated structures at a 0.01% or unquantifiable level (Aoki et al. 2007, Koles et al. 2007).

Although simple N-glycans (Man_5_GlcNAc_2_ and Man_3_GlcNAc_2_Fuc) have been predominantly observed throughout *Drosophila* embryogenesis, the exact profile of N-linked glycans is shown to be both spatially and temporally controlled (Aoki et al. 2007, 2008). This is indicative of stage and tissue-specific glycoprotein requirements and an associated regulation of glycosylation machinery, which can shift the balance between paucimannose and complex structures. More than 40 distinct glycoprotein species, all containing a paucimannose core, have now been identified in *Drosophila*, yet as observed in the earlier studies, these complex glycans are only present as minor components, with the vast majority remaining as unmodified high mannose or paucimannose structures. This is again distinct from mammals, where complex N-glycans with abundant sialylation are predominant. This invertebrate-specific abundance of paucimannose has been partially explained by the elucidation of a *Drosophila* hexosaminidase*—*β*-N*-acetylglucosaminidase, encoded by the gene *fused lobes* (*fdl*) (Aumiller et al. 2006, Leonard et al. 2006, Geisler et al. 2008). This enzyme removes GlcNAc residues that are added by *N*-acetylglucosaminyltransferase I (GlcNAcT-I), resulting in formation of paucimannose (and its fucosylated derivatives), whilst blocking progression to more complex glycans. Human isoenzymes (*HEXA* and *HEXB*) have been shown to drive paucimannosidic protein production in neutrophils (Ugonotti et al. 2022), through a noncanonical cascade, i.e. only proposed to occur in limited tissues and (patho)physiological conditions (Chatterjee et al. 2019, Parker et al. 2021)—unlike the constitutive and ubiquitous utilization of this pathway in invertebrates.

Several groups have utilized mass spectrometry to analyse glycoproteins in another well-characterized model organism, the nematode *C. elegans*; a body of work that has been reviewed in great detail by Paschinger et al. (2008). As with *Drosophila*, its well-characterized genetics helped identify candidate enzymes associated with the synthesis of hybrid and complex glycans; homologues of *N*-acetylglucosaminyltransferase I (Chen et al. 2002, Zhu et al. 2004), II (Chen et al. 2002), and V (Warren et al. 2002). Mass spectrometric analysis of *C. elegans* N-glycans has shown, as in *Drosophila*, an abundance of high-mannosidic class glycoproteins (Man_5–9_GlcNac_2_). Paucimannosidic structures (Man_3_GlcNAc_2_ Fuc_0–3_) are also copious in *C. elegans*, in which, as in *Drosophila*, the core can be fucosylated via α1–6 and α1–3 core linkages (Haslam et al. 2002, Paschinger et al. 2004, Cipollo et al. 2005, Natsuka et al. 2005, Hanneman et al. 2006). Despite the similarities, these studies also highlight several distinctive and unique features of *C. elegans* N-glycan species. For example, *C. elegans* glycan species can be fucosylated at, up to, three residues on the Man_2–3_GlcNAc_2_ core and five fucose residues on the mature glycan (Fig. 1iii) (Paschinger et al. 2019). More complex *C. elegans* glycans can link phosphorylcholine (PC) groups to a core or terminal GlcNAc. This modification is thought to be relatively frequent in the glycoproteins of *C. elegans* and other nematodes compared to other invertebrates (Stanton et al. 2017, Martini et al. 2019), and associated with immunomodulatory properties (Harnett et al. 1998, Pineda et al. 2014) and/or be related to nematode growth and development (Lochnit et al. 2005). Longitudinal studies in *C. elegans* have noted the N-glycan profile was distinct at each developmental stage studied, and an increased degree of N-glycan complexity and PC-presence in the L1 and Dauer stages—*C. elegans* stages associated with significant lifestyle changes (Cipollo et al. 2005). Roughly 150 different N-glycan species have been identified in *C. elegans* and, as with *Drosophila*, the relative proportion of higher order glycans is low, suggestive of a gene acting in a homologous way to the *Drosophila fdl*. Recent studies have shown that mutant *C. elegans* with a partial deletion of a β*-N*-acetylhexosaminidase (*hex-2*), produce proportionally less paucimannose (Gutternigg et al. 2007), although significant amounts are still detectable, indicating the existence of supplementary *C. elegans* β*-N*-acetylhexosaminidase genes (*hex-3, -4, -5*).

Considering the number of N-glycan structures identified, alongside the potential modifications, the structural N-glycan diversity in insects and nematodes is vast, as is the repertoire of associated roles and locations (cell surface, ion channels, adhesion, and extracellular matrix among others). In fact, apart from the lack of sialylation, structural diversity is reported as comparable to that of mammals (Walski et al. 2017). Furthermore, interspecies diversity is also clear. This is highlighted by a recent comparative study showing minimal overlap in the N-glycoprotein profiles from four phylogenetically diverse insecta; the flour beetle (*Tribolium castaneum*; Coleoptera), the silkworm (*Bombyx mori*; Lepidoptera), the honeybee (*Apis mellifera*; Hymenoptera), and the fruit fly *D. melanogaster* (Diptera) (Vandenborre et al. 2011). The relevance of this diversity is yet to be fully understood with many questions remaining on establishing synthetic pathways, determining the functional relevance of N-glycans, and understanding the spatio temporal control throughout a life cycle. Indeed, shifts in glycoconjugate expression could play an important role in determining species susceptibility to a range of glycoconjugate binding toxins.

## O-linked protein glycosylation

**Figure 2.**
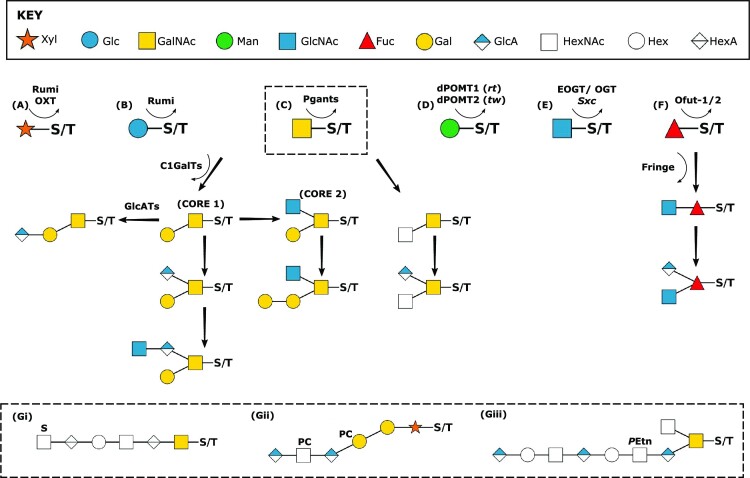
Overview of O-glycan diversity in insects and nematodes. The first residue attached to the serine/threonine determines the type of O-glycan (A) O-Xyl, (B) O-Glc, (C) O-GalNAc or mucin-type, (D) O-Man, (E) O-GlcNAc, and (F) O-Fuc. Mucin-type O-glycans (C) appear to be the most common glycans in studied insecta and nematodes with core 1 and core 1 modified with glucuronic acid (GlcA) generally most prevalent. Examples of *Drosophila* biosynthetic pathways illustrate some of the known O-glycan diversity with frequent sulphation and glucuronylation. (G) Examples of more complex structures and modifications including, (i) a sulphated (S) HexA-Hex-HexNAc repeat from Sf9 cells (Gaunitz 2013), (ii) a glycosaminoglycan-like zwitterionic glycan from the *Oesophagostomum dentatum* nematode modified with PC, and (iii) a Hex-HexNAc containing O-glycan modified with phosphoethanolamine (*P*Etn), present in both *Ae. aegypti* and *An. gambiae* larvae. Glycans are depicted according to the Symbolic Nomenclature for Glycans, as shown in **KEY**.

O-linked glycan diversity appears to be one of the most varied sets of posttranslational modifications across organisms and begins with the initial monosaccharide moiety linked to the (glyco)protein via the oxygen atom of serine or threonine (O-S/T). These initial monosaccharides can be O-Xyl, O-Glc, O-GalNAc (mucin-type), O-Man, O-GlcNAc, or O-Fuc (Fig. 2A–F). Mucin-type O-linked glycosylation appears to be the predominant form in *Drosophila* (the best-characterized insect species), for which the core structures and associated biosynthetic stages are conserved in vertebrates (as shown in Fig. 2C). Mucin-type glycans can be categorized by different core structures. In *Drosophila*, unmodified core-1 structures (Galβ1–3GalNAcα1-O-S/T or the ‘T-antigen’) are predominant (North et al. 2006). Core-1 structures modified with glucuronic acid (GlcA), core-2 structures (GlcNAcβ1–6(Galβ1–3)GalNAcα1-O-S/T), and a less well-characterized HexNAc–GalNAc core structure are also present in a comparatively reduced abundance (where Hex = any six carbon monosaccharide) (Aoki et al. 2008, Breloy et al. 2008). Lectin binding and mass spectrometry-based characterization of the O-glycan profiles in lepidopteran (Sf9 from *Spodoptera frugiperda*, Mb from *Mamestra brassicae*, and Tn from *Trichoplusia ni*) and dipteran (S2 from *D. melanogaster*) cell lines (Thomsen et al. 1990, Lopez et al. 1999), as well as larvae from two mosquito species (*Aedes aegypti* and *Anopheles gambiae*) (Kurz et al. 2015) have also all demonstrated a prevalence of mucin-type core 1 and 2 structures.

As with N-glycans, an extension of the core O-glycan structure to generate more complex patterning appears to be proportionally reduced in arthropods—in comparison to their mammalian counterparts (Fristrom and Fristrom 1982, Kramerov et al. 1996, Theopold et al. 2001, North et al. 2006). Further structural complexity and species-specific diversity is achieved through post synthetic modifications. For example, glucuronylated and sulphated O-glycans are observed in *Drosophila* (Breloy et al. 2008), *Ae. aegypti, An. gambiae*, and various lepidopteran cell lines (Fig. 2 Gi) (Garenaux et al. 2011, Gaunitz et al. 2013), and phosphoethanolamine is linked to HexNAc residues in wasps and mosquitoes (Fig. 2 Giii) (Garenaux et al. 2011, Kurz et al. 2015). Insect and nematode glycan diversity could also be heavily influenced by the environment. Indeed, cell media composition has been indiciated to influence the O-glycosylation potential of a range of insect cell lines significantly (Lopez et al. 1999), and an upregulation of mucins (a glycoprotein class where >50% have O-glycosylation), has been reported in the nematode *Laxus oneitus* under conditions of anoxia (Paredes et al. 2022). The exact role of the environment and substrate scavenging in the role of insect glycan synthesis remains to be determined.

Our understanding of the most common O-glycans (O-GalNAc, mucin-type) has been significantly aided through the elucidation of 14 putative *Drosophila* UDP–GalNAc:Polypeptide *N*-acetylglucosaminyltransferases (pgants)–homologs of the mammalian enzymes required for the initial transfer of GalNAc from the UDP–GalNAc to the Ser/Thr hydroxyl group (Gerken et al. 2008, Ten Hagen et al. 2003a, b). Biochemical analysis has shown functional conservation between mammalian and *Drosophila* orthologues with some pgants acting as glycopeptide transferases (GalNAc modified substrate) and others as peptide transferases (unmodified peptide substrate). Additionally, *pgant* genes are shown to be spatially and temporally regulated throughout *Drosophila* development, suggesting a distinct regulation of O-glycan patterning (Tian and Ten Hagen 2006). Demonstrating the functional importance of appropriate O-glycosylation, *pgant35A Drosophila* mutants show embryonic, larval, and pupal lethality—the first demonstration of O-linked mucin-type glycosylation being essential for viability (Ten Hagen and Tran 2002, Schwientek et al. 2002b). Further studies with *pgant35A* maternal mutants showed reduced localization of mucin-type glycans on the apical and luminal surfaces of the developing respiratory system and a loss of tracheal integrity (Tian and Ten Hagen 2007). Lethality is also observed in *Drosophila* that cannot generate the core-1 T antigen—(C1GalTa enzyme mutants)—potentially due to abnormalities in CNS morphogenesis (Lin et al. 2008, Xia et al. 2004).

Alternative O-linked structures (O-Man, O-Glc, O-GlcNAc, O-Fuc, and O-Xyl; Fig. 2) have been detected in *Drosophila* (Kurz et al. 2015), mosquitoes (Kurz et al. 2015), nematodes (Vanbeselaere et al. 2018), lepidopteran cell lines (Lopez et al. 1999), and hymenopteran tissues (Garenaux et al. 2011), demonstrating divergent structures with distinct tissue distributions. Genetic studies investigating the effects of reduced transferase activity have repeatedly demonstrated the importance of this, more minor, glycan patterning (Kelly and Hart 1989, Ju and Cummings 2002, Okajima et al. 2003, Ten Hagen 2003a, b) and the conservation of functional pathways between eukaryotes. For example, *Drosophila* have two orthologues of the vertebrate O-mannosyltransferases (dPOMT1 and dPOMT2), encoded by *rotated abdomen (rt)* and *twisted, (tw)*, which are both required for the mannosylation of protein substrates (Ichimiya et al. 2004, Lyalin et al. 2006). Mutations in either *Drosophila rt* or *tw*, causes defective muscular development and, as the name suggests, a rotated abdomen phenotype. In humans, mutations in *Pomt* genes are associated with muscular dystrophies (Muntoni et al. 2004a, b), highlighting the functional similarities of vertebrate and insect O-glycans. As another important example, O-linked fucose (and elongated b3-linked GalNAc generated via *Fringe*) residues are shown to play critical roles in embryonic development in insects and mammals through the glycosylation of Notch receptors and subsequent modification of Notch receptor ligand preferences (Okajima and Irvine 2002, Okajima et al. 2003, Sasamura et al. 2003, Pandey et al. 2019). O-Xyl modification of serine residues represents the first stage in the synthesis of glycosaminoglycan (GAG)-like O-glycans—linear polysaccharides consisting of a repeating two sugar-unit consisting of a six-carbon acidic sugar (HexA) and an amino sugar (HexNAcHexA)_n_. Nematodes, *C. elegans* and *O. dentatum*, have shown conservation of the common mammalian tetrasaccharide core (GlcAβ1–3Galβ1–3Galβ1–4Xylβ-O-Ser) (Yamada et al. 1999, Guerardel et al. 2001), and also shown the addition of galactose and PC (Vanbeselaere et al. 2018). These nematode GAGs are demonstrated to be important for development, with the mutation of *C. elegans* xylosyltransferases (*sqv-2* and *sqv-6*) inhibiting GAG biosynthesis, altering vulval morphogenesis and zygotic cytokinesis, and maternal-effect lethality (Hwang et al. 2003). GAG-like glycans have also been identified in *Drosophila* (Yamada et al. 2002), and have been associated with development and facilitating pathogen invasion (Park et al. 2003, Baron et al. 2009).

As with N-glycans, the elucidation of currently unknown insect biosynthetic enzymes will help us to dissect the molecular function of O-glycans and the relevance of various structural features.

## Glycolipids in insects and nematodes

**Figure 3.**
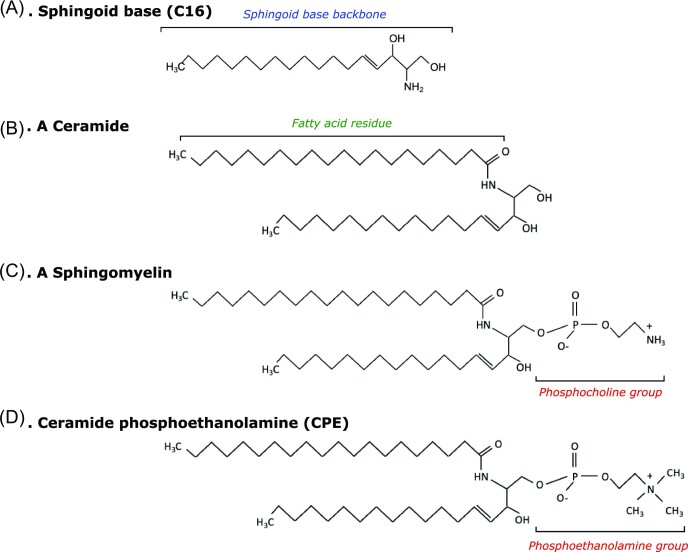
General structures of Sphingolipids. Sphingolipids are a class of lipids, which contain a backbone of sphingoid bases which is N-acylated with various fatty acid chains. (A) A sphingoid base composed of a 16-carbon backbone (C16, hexadecanoylsphinganine). (B) A ceramide, consisting of a sphingoid base backbone amide linked to a fatty acid. (C) A sphingomyelin, a phosphocholine headgroup attached to a ceramide. (D) Ceramide phosphoethanolamine (CPE), a phosphoethanolamine headgroup attached to a ceramide.

Glycolipids are lipids with a carbohydrate attached via a glycosidic bond, with known roles in maintaining cellular membrane integrity, facilitating cell-to-cell and intracellular signalling, initiating host immune responses, and determining blood groups. GSLs are a subclass of glycolipid where the carbohydrate group is covalently attached to a ceramide backbone moiety (a sphinganine, i.e. amide linked to a fatty acid; Fig. 3). GSLs are of particular interest when considering potential receptor functions, as they are known toxin receptors (Geny and Popoff 2006), and found enriched in cellular membrane microdomains (lipid rafts) that act as specialized platforms for signal transduction and protein/lipid transport (Simons and Ikonen 1997, Brown and London 1998).

Initial investigations into insect GSLs in 1973 by Luukkonen et al. (1973), showed an absence of complex GSLs in cells cultured from *Aedes albopictus*. However, later reports identified the first GSLs in arthropods, by utilizing 2D high-performance thin-layer chromatography (HPTLC) to indicate the presence of glucosylceramide (GlcCer) and mannosyl-glucosylceramide (Man-GlcCer) in two closely related dipteran species; the larvae of the green-bottle fly, *Lucilia caesar*, and the pupae of the blowfly, *Calliphora vicina* (Sugita et al. 1982a, Dennis et al. 1985b). This was followed by several ground-breaking studies from Sugita, Hori, Dennis, Wiegandt and others, predominantly in the same dipteran species, showing arthropods form an ‘arthro-series’ of GSLs derived from a single, neutral, Manβ1,4Glcβ-ceramide core—termed mactosylceramide (MacCer) (Sugita et al. 1982a, b, 1989 , 1990, Dennis et al. 1985a, b, Dabrowski et al. 1990, Weske et al. 1990, Helling et al. 1991). This invertebrate-specific glycolipid signature is conserved in nematodes and insects but is divergent from vertebrates, where the majority of GSLs are derived from a lactosylceramide core (LacCer; Galβ1,4Glcβ-ceramide). Using a combination of HPTLC, sequential exoglycosidic digestion, methylation analysis, and direct-inlet mass spectrometry (MS), these aforementioned studies in dipteran insects went on to find neutral, acidic, and zwitterionic GSLs with increasing complexity and oligosaccharide length—all as extensions of the MacCer core. Dipteran GSLs were also identified to be frequently modified with phosphoethanolamine (*P*Etn) linked to C6 of GlcNAc, resulting in a zwitterionic core structure.

**Figure 4.**
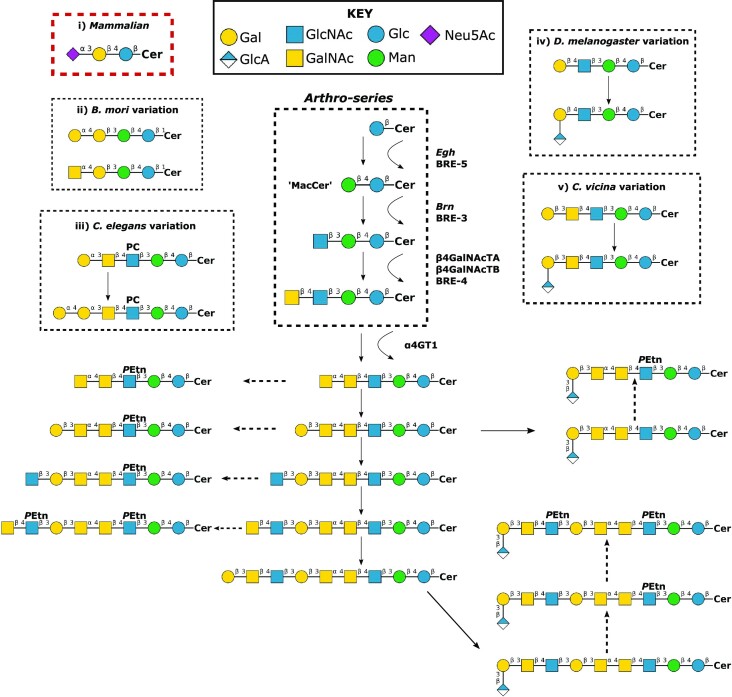
Overview of GSL synthesis and diversity in insects and nematodes. Arthroseries GSLs present in insects and nematodes are built around a common MacCer core, generated through addition of Man to a glucosylceramide, in contrast to the mammalian GlcCer core, exampled in GM3 (i, red box). This core can be extended to form more complex structures, such as those shown above, which have all been identified in *Drosophila* embryos (and in some cases, other Diptera). *Drosophila* figure components modified from Sharrow et al. (2010). Modification with phosphoethanolamine (*P*Etn) on GlcNAc residues is frequent and generates zwitterionic GSLs, whilst addition of GlcA to terminal Gal residues generates acidic groups. Some of the known glycosyltransferases that facilitate GSL biogenesis are marked; *Drosophila* (*Egh, Brn*, α4GT1, β4GalNAcTA, and β4GalNAcTA) and *C. elegans* (BRE-5, BRE-3, and BRE-4). Although the structures in the ‘Arthro-series’ box with the sequential addition of GlcNAc then GalNAc appear to be the most common root of more complex GSLs across invertebrate species, species-specific variants are frequently noted, such as those depicted in (ii) *B. mori* (Itonori 2018), (iii) *C. elegans* (Griffitts 2005), (iv) *Drosophila* (Aoki and Tiemeyer 2010), and (v) *C. vicina* (Dennis et al. 1985a). PC = phosphorylcholine, glycans are depicted according to the Symbolic Nomenclature for Glycans as shown in **KEY**.

*Drosophila melanogaster* has become the predominant choice for studying arthropod GSLs, with the biosynthesis pathways and structural variants now relatively well-understood (Fig. 4)—as summarized in greater detail by Aoki and Tiemeyer (2010). Analysis of *Drosophila* GSLs indicated the presence of a similar family of variants to that observed previously in *L. caesar* and *C. vicina* (Fredieu and Mahowald 1994, Callaerts et al. 1995, D’Amico and Jacobs 1995, Seppo et al. 2000). However, there are noted *Drosophila* distinctions such as an increased proportion of longer GSLs that are substituted with two *P*Etn residues (Itonori et al. 2005, Aoki and Tiemeyer 2010), and a 4-linked GalNAc (as opposed to a 3-linked GalNAc) in the longest characterized *Drosophila* GSL (Seppo et al. 2000). Studies in other insects and nematodes have also indicated that a distinct species-specific GSL diversity is present (Fig. 4i–iv). For example, although the MacCer core is most commonly extended with GlcNAc via a β1–3 linkage followed by GalNAc via a β1–4 linkage, *Drosophila* can extend with Gal, rather than GalNAc, followed by Glucuronic acid (GlcA) (Fig. 4i) (Aoki and Tiemeyer 2010). Additionally, the later steps of biosynthesis appear to diverge between dipterans (*Drosophila* and *Calliphora*) and nematodes. In both these dipteran genera, the common core tetrasaccharide (GalNAcβ1–4GlcNAcβ1–3Manβ1–4Glcβ-Cer) is extended by a GalNAc, whereas *C. elegans* extends with an α1,3-linked Gal. Furthermore, the core GlcNAc can be substituted with PC (Fig. 4iii)—a modification that appears to be conserved in parasitic nematodes (Gerdt et al. 1999, Wuhrer et al. 2000). Whether these distinctions always reflect true species-specific GSLs or developmentally regulated expression in the material studied (embryonic, larvae, or pupae) is not completely clear. Indeed, GSL synthesis is highly regulated in mammals—both spatially and temporally—with dysregulation prevalent in disease such as storage disorders (Breiden and Sandhoff 2019) and cancers (Furukawa et al. 2019). The ability to diversify functional lipids significantly, early in the biosynthesis pathway, may tailor GSLs for specific spatial or temporal functions—such as development or toxin binding in localized regions of the insect gut. Temporal artificial manipulation of GSL biosynthesis may be a useful approach for investigating toxin binding at different stages in an insect’s life cycle. For example, many mammalian studies have utilized small molecule inhibitors of glycolipid biosynthesis pathways, and different cell culture media additives are known to drastically alter cellular glycosylation profiles.

The presence of insect gangliosides (GSLs that contain one or more sialic acid residue) remains controversial, as reviewed previously (Ghosh et al. 2018, Marchal et al. 2001). Whilst little is known about insect sialylation, eukaryotic sialylation is well-studied and has diverse roles in development of the central nervous system, immune response, cell death, cell signalling pathways, host–virus interaction, as well as pathogenic implications in Alzheimer’s disease and cancer progression (Varki et al. 2008, Schauer 2009, Ghosh et al. 2015, Yanagisawa et al. 2015, Teppa et al. 2016). Sialic acids, sialylated macromolecules and sialyltransferase (ST) enzymes have been reported in a range of insects including *B. mori* (Kajiura et al. 2015), *D. melanogaster* (Koles et al. 2004), *Ae. aegypti* (Cime-Castillo et al. 2015, Di et al. 2017), and *Galleria mellonella* (Karacali et al. 1997) but, despite this, insect investigations indicate that gangliosides do not appear to be intrinsically present at a detectable level (Aoki et al. 2007, Koles et al. 2007). Additionally, little is known about the synthesis or function of sialic acid moieties, and there is no structural information surrounding STs. Arthro-series GSLs capped with GlcA on a nonreducing terminal are common and have been identified in flies (*C. vicina* and *D. melanogaster*) (Wiegandt et al. 1992). GlcA carries a negative charge under physiological conditions, prompting comparisons to the sialic acid-containing gangliosides of vertebrates and the term ‘arthrosides’. Currently, there are very limited data to support a functional comparison. Furthermore, sialic acids can be α2–8 linked to additional sialic acids whereas GlcA dimers, to the best of our knowledge, have not been reported.

In addition to the sugar component of GSLs, it must also be noted that the ceramide (a sphingoid base backbone linked to a fatty acid) backbone composition also differs between invertebrates and mammals. Mammalian sphingoid bases tend to be longer (generally C18) (Sullards et al. 2003), whereas insect sphingoid bases are generally reported as C14 and C16 and are amide linked to shorter fatty acid chains (Oswald et al. 2015) (Fig. 3A and B). In many arthropods, ceramide phosphoethanolamine (CPE) is the bulk sphingolipid (Fig. 3D) (Panevska et al. 2019), whereas only trace amounts of CPE have been detected in mammalian cells (Bickert et al. 2015) and Nematoda (Satouchi et al. 1993) which, instead, favour sphingomyelin synthesis (a ceramide with a phosphocholine group; Fig. 3C). Distinct biophysical properties have been observed between sphingomyelin and CPE in terms of membrane-order parameters (Terova et al. 2005, Bjorkbom et al. 2010) and the ability to interact with cholesterol and form lipid-rafts (Ramstedt and Slotte 2006, Bjorkbom et al. 2010), suggesting they have differing biological roles (Dawaliby et al. 2016). It may be that these GSL backbone differences play a part in determining binding specificity of insecticidal proteins, yet, to the best of our knowledge, this has not been investigated.

As with vertebrates, the complexity of insect and nematode GSLs occurs along common biochemical pathways via specific, glycosyltransferase-catalyzed, sequential addition of monosaccharides. Elucidation, and manipulation, of these glycosyltransferases has provided an insight into GSL function and utility. The first committed step in GSL synthesis is through the addition of glucose to ceramide via glucosylceramide synthase (GlcCer). Knockdown of an embryonic *Drosophila* GlcCer homolog caused increased apoptosis, indicating a requirement for GSLs—at least during development (Kohyama-Koganeya et al. 2004). Catalyzing the second and third steps in *Drosophila* GSL synthesis are two genes *brainiac (brn)* and *egghead (egh)*—initially proposed to act in the same functional pathway based on similar developmental phenotypes exhibited by their respective mutants—namely an over proliferation of neural cells and enlarged peripheral nerves. The *brn* gene was determined to encode a β1,3GlcNAc transferase directed to transfer GlcNAc preferentially to the Manβ1,4Glc core structure (Muller et al. 2002, Schwientek et al. 2002a), and *egh* to encode a β1,4-mannosyltransferase to form MacCer (Fig. 4) (Wandall et al. 2003). Both *Brn* and *egh* mutants are lethal, implying a requirement for second and third step sugar addition. Interestingly, inhibiting the fourth step in GSL synthesis—via null mutation of β1,4*-N*-acetlygalactosaminyltransferases (β4GalNAcTB/β4GalNAcTA) is not lethal, although still causes defects including the ventralization of ovarian follicle cells (Chen et al. 2007). *Drosophila* α1,4-*N*-acetylgalactosaminyl transferase (α4GTI) synthesizes the ceramide–pentahexoside (Mucha et al. 2004), although as fourth step (β4GalNAcTB/β4GalNAcTA) mutants are still viable, this is also presumably nonessential for viability. Toxicity studies in the nematode *C. elegans* (discussed in greater detail below) have found genes homologous to *brainiac* and *egghead, bre-5*, and *bre-3*, respectively.

As with N and O glycans, it is clear that an increasing range of glycolipid structural variants is being identified in insects and nematodes, even if these more complex structures do not make up the majority of the total pool. Key to deciphering the molecular function of these glycoconjugates is the elucidation of glycosyltransferases. Altering glycolipid biosynthesis pathways—through manipulation of glycosyltransferase activity via gene silencing or inhibitory compounds—will help to inform approaches towards current, and novel, methods of pest control.

## Glycoconjugates as membrane receptors for insecticidal and nematocidal toxins

The role of host cell membrane glycoconjugates as toxin receptors has ample precedent (Zuverink and Barbieri et al. 2018). Toxins that rely on glycoprotein binding include pertussis toxin (Stein et al. 1994) and aerolysin (Diep et al. 1998). Examples of protein toxins shown to use lipid-moieties to facilitate entry include the pore-forming toxins lysenin (via sphingomyelin (Yamaji et al. 1998) and cholesterol-dependent cytolysins (Tweten et al. 2005), Shiga toxin (via GSL Gb3; Okuda et al. 2006, Shin et al. 2009), and cholera toxin (via GM1a ganglioside; Wernick et al. 2010). Lipid microdomains are also implicated in toxin binding due to the high concentration of GSLs present. For example, cholera toxin-induced membrane curvature is shown to be dependent on both the multiplicity and specific geometry of GM1a binding sites (Kabbani et al. 2020), and Shiga toxin is localized to Gb3 in lipid rafts (Smith et al. 2006). Some toxins, such as members of the Botulinum toxin family, utilize both a ganglioside and a protein receptor, whereas others, such as ricin, bind a specific carbohydrate moiety that can be present on either a glycolipid or a glycoprotein (Fu et al. 1996, Zuverink and Barbieri 2018). Below we will discuss the existing research surrounding the role of glycoconjugates in insecticidal and nematocidal 3D-Cry protein toxin activity. Lectins are carbohydrate-binding proteins which are, individually, highly specific to a distinct sugar group (Cummings and Etzler 2009). Lectins have been incredibly useful, and widely used, in elucidating the sugar binding properties of various insecticidal toxins; those discussed in this review are summarized in Table 1.

**Table 1.** Specificity of lectins used commonly in lectin binding studies.

Lectin	Major sugar specificity
Wheat germ agglutinin (WGA)	GlcNAc (Gallagher et al. 1985)
Concanavalin A (ConA)	Man > αGlc, αGalNAc (Osawa and Tsuji 1987)
*Galathus nivalis (*GNA)	Man (α1,3 > α1,6 > α1,4)* (Hester and Wright 1996)
*Aleuria aurantia* (AAA)	Fuc (α1,6 > α1,3 > α1,4)* (Yamashita et al. 1985)
*Arachis hypogea* Peanut (PNA)	Galβ1–3GalNAcα1,3-Ser/Thr (Chacko and Appukuttan 2001)
Soybean agglutinin (SBA)	GalNacα1,3-Ser/Thr, (Sueyoshi et al. 1988)
*Ulex europaeus* agglutinin I (UEA1)	α-linked fucose (Tian et al. 2018)
*Datura stramonium* (DSA)	Galβ1,4GlcNAc (Crowley et al. 1984)

* > denotes the binding affinity where a single lectin can bind different linkages.

Several of the studies, discussed below, utilize cellular models to investigate 3D-Cry binding affinity and toxicity. In these studies, it is worthwhile to consider the impact of pH, as 3D-Cry proteins are solubilized and activated in the midgut lumen due to selective pH conditions (Knowles et al. 1994). In the literature, the insect midgut is often referred to as alkaline—a characteristic, i.e. often cited to assist in conferring insect species selectivity. Indeed, the majority of Dipteran and Lepidopteran species assessed have an alkaline midgut (~pH 8.0–10.0), although there are exceptions such as *Marasmia trapezialis* (pH 7.0–7.2), *Pieris rapae* (pH 7.3–7.6), and *Corcyra cephalonica* (pH 7.0–7.6) (Berebaum et al. 1980). Furthermore, there are often differences between the posterior and anterior midgut regions, such as *Ae. aegypti* and *Aedes canadensis* mosquito larvae (~pH 8 in the gastric caecum, > pH10 in the anterior midgut, pH 7.5 in the posterior midgut) (Dadd et al. 1975, Boudko et al. 2001). In contrast, other insects can have a mildly acidic midgut such as Coleoptera, *Leptinotarsa decemlineata* (pH 6.5–5.36) (Krishnan et al. 2007) and *Diabrotica virgifera virgifera* (pH 5.75) (Kaiser-Alexnat 2009). In terms of cell culture experiments the pH will be determined by buffer or culture media (which are frequently more acidic than mammalian media,~pH 6.2–6.5). In many experiments the toxin in question is solubilized and activated before addition to cells, via extracted ‘midgut-juice’ or artificially with buffer and proteinases—which in theory should negate the need for ‘mid-gut’ conditions for solubilization and activation but may alter the binding affinities via protonation states of key residues.

## Cry1A (Cry1Aa, Cry1Ab, and Cry1Ac)

### Binding to BBMVs show Cry1Ac binds in a GalNAc-dependent manner

The Cry1A subclass of lepidopteran-specific toxins are of great commercial importance and the most well-studied 3D-Cry toxins. The earliest glycoconjugate binding studies were performed using endotoxin isolated from Bt serovar. *kurstaki* HD-1 (Btk HD-1), which was later confirmed to contain three distinct Cry1A proteins that share >76% aa identity as protoxins; Cry1Aa, Cry1Ab, and Cry1Ac (Hofte and Whiteley et al. 1989). These early studies proposed the occurrence of a common Cry insecticidal pore-forming action (Hofmann et al. 1988a, b), yet identified mechanistic heterogeneity dependent on individual Cry proteins, target species, and putative binding ‘receptors’. Of note, early studies using the Btk HD-1 strain also likely contain other Cry proteins including Cry2Aa2, Cry2Ab2, and Cry1Ia3.

The relevance of glycoconjugates in eliciting toxin activity was recognized early on, with Knowles et al. (1984) showing that GalNAc and GlcNAc binding-lectins (SBA and WGA, respectively) neutralized activity of lepidopteran-active δ-endotoxin proteins from Btk strain HD-1 in a lepidopteran cell line (CF1) isolated from the Cry1A-susceptable cabbage butterfly (*Choristoneura fumiferana*). Using the same model, they went on to identify the first putative Cry ‘receptor’—a 146-kDa cell-surface glycoprotein capable of binding both SBA and δ-endotoxin (Knowles and Ellar 1986). Dennis et al. (1986) first proposed that glycolipids were responsible for modulating δ-endotoxin actions, through demonstrating Btk HD-1 toxin binding to distinct *C. vicina* pupal GSLs—of which some species contained a relevant terminal GalNAc residue. In these studies, they isolated both total neutral and total acidic glycolipid fractions, and isolated neutral GSL components that they probed using a thin layer chromotography (TLC) overlay technique to detect binding of both the protoxin and activated forms of Btk HD-1 proteins. Although Btk HD-1 contains a number of toxins (Yamamoto and McLaughlin 1981a), the authors only used the ∼130 kDa proteins—most likely representing a mix of Cry1 proteins. Multiple binding partners were observed in both glycolipid fractions, with the main component (bound by both the protoxin and activated forms) being Galα1–3GalNAcβ1–4GlcNAcβ1–3Manβ1–4Glcβ1–4Cer (denoted as 5B by the authors). Although both the protoxin and activated form were shown to bind strongly to the Gal-terminal 5B glycolipid, the toxin showed a decrease in binding specificity after activation, with an increased number of glycolipids bound and an increase towards glycolipids with terminal GalNAc residues. Different binding patterns between the pro and active forms would indicate the binding of protoxin would not block activity of the activated protein through competition for binding. When reading these works, it is important to consider that these binding experiments utilized models containing cells derived from nontarget tissues, which potentially present glycoconjugates found predominantly outside of the midgut, and in an altered abundance. Brush border membrane vesicles (BBMVs) prepared from larval midguts provided a more ‘*in vivo’* representation and became common in the field for investigating toxin binding to apical microvilli. Using BBMVs or gut tissues, isolated from a range of lepidopteran species, several investigations confirmed a range of specific Cry1A binding sites with nM affinity constants (Jaquet et al. 1987, Van Rie et al. 1989, 1990, Wolfersberger et al. 1990, Ferre et al. 1991, Garczynski et al. 1991, Denolf et al. 1993). In many cases the level of Cry1A toxicity was shown to correlate with binding affinity (Hofmann et al. 1988b; Van Rie et al. 1989, 1990, Garczynski et al. 1991, Denolf et al. 1993). For example, Cry1Ab and Cry1Ac recognize the same receptor on *Ostrinia nubilalis* BBMV, yet the former has an 11-fold higher affinity which correlates with a 10-fold higher toxicity (Denolf et al. 1993). The importance of these binding sites was further illustrated by work in a field population of *Plutella xylostella*, where resistance to Cry1Ab was associated with loss of BBMV binding sites (Ferre et al. 1991). Furthermore, these studies illustrated frequent receptor heterogeneity and the existence of multiple binding sites, with increased binding site concentration also associated with increased toxicity (Van Rie et al. 1989, 1990, Garczynski et al. 1991). For example, *H. virescens* larvae show three different populations of binding site, one which binds Cry1Aa, Cry1Ab, and Cry1Ac, a second which binds Cry1Ab and Cry1Ac, and a third restricted to Cry1Ac binding. This correlates with the pronounced larvicidal difference between Cry1A variants (Ac > Ab > Aa) (Van Rie et al. 1989, 1990). Receptor proteins originally identified from ligand binding studies in BBMV have since been purified and characterized. Two major forms of putative Cry receptor have been identified, namely cadherin-like receptors (CAD) (Vadlamudi et al. 1993, 1995), and aminopeptidase-N (APN) family receptors (Knight et al. 1994, Sangadala et al. 1994)—both shown to be glycosylated. Other receptor families for insecticidal toxins include alkaline phosphatase (ALP) (Jurat-Fuentes and Adang 2004, McNall and Adang 2003, Krishnamoorthy et al. 2007, Arenas et al. 2010, Ning et al. 2010) and ATP-binding cassette (ABC) transporter protein (Xiao et al. 2014, Guo et al. 2015, Chen et al. 2018. Wang et al. 2019, Wu et al. 2019). Roles for putative glycosylation sites in the latter two receptor families are less well-explored—with no specific role for glycosylation reported for Cry1 ABC receptors.

However, toxicity does not always correlate with BBMV protein binding (Van Rie et al. 1990, Wolfersberger et al. 1990, Ferre et al. 1991, Garczynski et al. 1991). This is exemplified by Garczynski et al. (1991), showing similar high affinity Cry1A binding to BBMVs isolated from both highly susceptible (*Manduca sexta* and *H. virescens*), moderately susceptible (*Helicoverpa zea*), and tolerant (*S. frugiperda*) lepidopteran larvae. Kumaraswami et al. (2001), and Higuchi et al. (2007), demonstrated BBMV proteins isolated from either susceptible or resistant populations of *P. xylostella* have the same Cry1A binding capacity, yet resistant insect-derived BBMV and gut tissue had a significant reduction in neutral GSLs, indicating these glycolipids can mediate toxin susceptibility. In resistant *P. xylostella* populations, this was accompanied by decreased oligosaccharide length, with synthesis arrest at the pentasaccharide stage and a slightly reduced activity of Gal and GalNAc transferase, suggesting that more elaborate glycolipid moieties facilitate Cry1A toxicity (Kumaraswami et al. 2001). More recent work by Ma et al. (2012a), supports the role of glycolipids in Cry1Ac binding and tolerance. *Helicoverpa armigera* larvae demonstrate enhanced tolerance to Cry1Ac if they are prefed with LEC-8–a galectin-like protein isolated from nematodes. Both LEC-8 and Cry1Ac were shown to bind to gut glycolipids in a similar manner, implying that LEC-8 inhibits Cry1Ac glycolipid binding sites, thus mediating tolerance. The LEC-8 natural ligand is unknown, but an inhibitory ELISA showed lactose can inhibit LEC-8 binding to *H. armigera* gut glycolipids by 20%, and a mild inhibitory effect was observed with GalNAc, galactose, mannopyranose, inositol, and trehalose. LEC-8 has also been shown to interact with Asialofetuin—a glycoprotein with terminal GalNAc residues (Nemoto-Sasaki et al. 2008).

Differences in neutral sugar content between susceptible and resistant *M. sexta* populations has been reported to correlate with Cry1A binding by a number of groups (Sangadala et al. 2001, Jurat-Fuentes et al. 2002). Knowles et al. (1991), solidified a role for a glycoconjugate in Cry1A binding in insect gut epithelia. GalNAc addition completely abolished Cry1Ac binding in *M. sexta*, partially in *H. virescens*, but had no effect on *Pieris brassicae*. This correlated with SBA and Cry1Ac binding the same (glyco)protein in *M. sexta* and *H. virescens*, but not *P. brassicae*, collectively indicating GalNAc is a component of the Cry1Ac receptor(s) in some lepidopteran species, but glycoprotein interaction is not required in others e.g. *P. brassicae*. The authors did not investigate the possibility of binding to GalNAc present in glycolipids. Although much of the literature to date is focused on the role of GalNAc in eliciting Cry1Ac toxicity, Haider and Ellar (1987) have proposed the relevance of D-Glc in eliciting Cry1 activity. Here, the authors showed the activity of a trypsinized lepidopteran-specific preparation from *Bt* serovar. *aizawai* IC1 (containing a 55- and a 58-kDa polypeptide) is completely inhibited in *M. brassicae* cells by D-Glc and the D-Glc binding lectin—ConA. It is not clear exactly what protein toxins were expressed in this preparation, although Cry1Ab7 is reported in this strain (Haider and Ellar 1988).

Conversely, glycolipid and sugar binding is also implicated in enhancing tolerance to Cry toxins through the sequestration of toxin oligomers in the gut and subsequent prevention of receptor binding in the midgut brush border (Hayakawa et al. 2004, Ma et al. 2012a, b). The peritrophic membrane (PM) is the semipermeable lining of the insect midgut which, among its functions, acts as protection from mechanical and pathogenic damage. Several studies have indicated that compromising the integrity of the PM can enhance Bt toxin activity in insect larvae, presumably through allowing more insecticidal protein to reach receptors at the midgut epithelium brush border (Granados et al. 2001). Hayakawa et al. (2004), demonstrated that the interaction of Cry1Ac with the PM can be inhibited with the addition of GalNAc in the Cry1Ac tolerant lepidopteran species, *B. mori*. Upon addition of GalNAc, Cry1Ac passes through the PM significantly quicker, and at a similar rate to the *B. mori* active toxin, Cry1Aa—although the authors did not demonstrate if this renders *B. mori* Cry1Ac susceptible. Ma et al. (2012b), have suggested that binding of Cry toxin to glycolipids in lipophorin (lipoprotein particles that transport lipids in insect haemolymph) increases Cry toxin tolerance. They demonstrated that D-II of Cry1Ac monomers binds glycolipids from lipophorin particles, and forms Cry1Ac oligomers in the presence of glycolipids isolated from both *H. armigera* and *G. mellonella* cell-free plasma and midgut tissue. Cry1Ac addition to *G. mellonella* lipid particles induced aggregation—an interaction through which, the authors suggest, Cry1Ac is sequestered to the gut lumen. This study also used TLC to show the main Cry1Ac glycolipid binding species present in *H. armigera* gut tissue migrated to a similar position as globoside Gb4 (GalNAc_3_β1–2Galα1–4Galβ1–4Glcβ1–1-Cer)—which has a terminal GalNAc.

The exact mechanistic basis for Cry1A toxicity remains unclear. A large body of data shows insecticidal activity is dependent on much more than a single receptor interaction, but with the exact insect system, toxin oligomerization state, multicomponent complexes, and tissue localization all having profound effects on toxicity. The most established mechanism for Cry1A appears to be that of sequential binding during which a toxin monomer is recognized by a cadherin-like receptor causing a conformational change, which facilitates prepore oligomer formation (and distinct types of prepore may be possible even for the same toxin; Gomez et al. 2014), and the subsequent binding to APN enabling membrane insertion. Multiple and complex receptor binding is not uncommon in the toxin field outside of 3D-Cry proteins, e.g. diphtheria (Hasuwa et al. 2001) and protective antigen (Scobie et al. 2003) are determined to utilize more than one receptor. Furthermore, as discussed in the introduction, the role of the prodomains in toxicity is yet to be fully elucidated. Aside from the commonly hypothesized roles in toxin stability, formation, and stabilization (Derbyshire et al. 2001), the structure of Cry1Ac1 protoxin D-V and D-VII have four predicted ligand binding sites for galactose, *N*-acetylglucosamine, mannose, and xylose (Zghal et al. 2017), presenting the possibility that D-V and D-VII could interact with glycans in the gut, and may be involved in protoxin recognition of a receptor. In support of this idea, a recent study by Peña-Cardeña et al. (2018), has demonstrated the C-terminal protoxin domain of Cry1Ab provides additional binding sites for ALP and APN receptors, resulting in a higher binding affinity of the protoxin, which correlates with increased toxicity—compared to the activated form.

### APN and APN glycosylation in mediating Cry1A binding and activity

Utilizing protoxin affinity chromatography and anion-exchange chromatography, (Knight et al. 1994), purified a glycoprotein (APN1) present in the midgut target tissue of *M. sexta* that was bound by Cry1Ac and SBA, but not Cry1B. Sequencing of the bound glycoprotein revealed sequence similarity to the APN family—a heavily glycosylated zinc aminopeptidase, i.e. a common feature of the insect midgut and, therefore, often used to assess BBMV purity. APNs have since been extensively studied as Cry receptors and many different lepidopteran variants have been characterized—although not all bind Cry proteins. APNs are divided into eight phylogenetic classes (Crava et al. 2010, Hughes et al. 2014, Fonseca et al. 2015), with single insect species able to express multiple receptors from different classes. APN isoforms that bind Cry1Aa (Masson et al. 1995), Cry1Ab (Masson et al. 1995, Denolf et al. 1997), and Cry1Ac (Gill et al. 1995, Valaitis et al. 1995, Wang et al. 2005b, Luo et al. 1997, Nakanishi et al. 2002, Angelucci et al. 2008) have been discovered in multiple lepidopteran species—although current evidence suggests only Cry1Ac binds via GalNAc, recognized by moieties present in a surface cavity in D-III, i.e. not conserved in Cry1Aa or Cry1Ab (Burton et al. 1999, de Maagd et al. 1999b, Jenkins et al. 2000, Masson et al. 1995). Putative Cry toxin receptors have been identified in APN classes 1–5, although recently APNs from classes 6 and 8 have been implicated in mediating toxicity of Cry1Ab, Cry1Ac, and Cry1Ca in *Chilo suppressalis* larvae (Sun et al. 2020).

**Figure 5.**
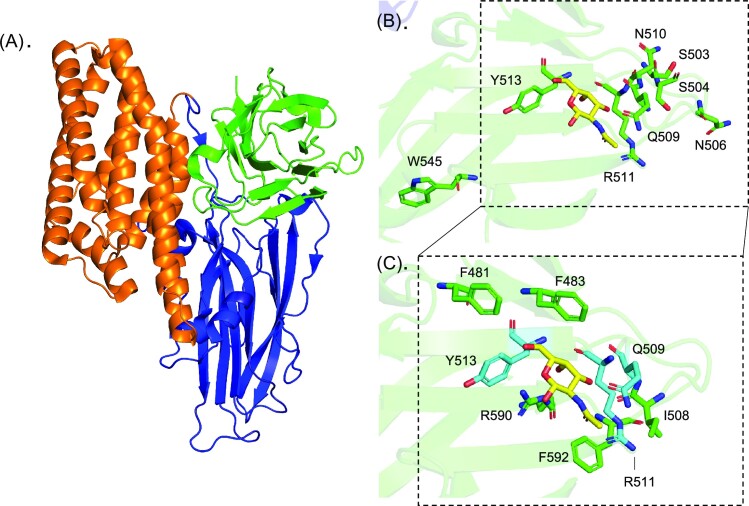
Crystal structure of Cry1Ac in complex with GalNAc (PDB 4ARY) and a summary of notable residues identified through mutational and crystallography studies. (A) Cry1Ac shares the conserved three-domain structure of the Cry family of toxins. Domain I (orange) comprises an α-helix bundle, domain II (blue) comprises three β-sheets forming a β-prism, and domain III (green) comprises two antiparallel β-sheets forming a ‘jellyroll’ domain. (B) Residues (shown as sticks) implicated as significant for APN binding and/or Cry1Ac toxicity against *L. dispar, M. sexta*, and *H. virescens* in Domain III. The binding of GalNAc (yellow) relative to these residues is also shown. (C) Residues that interact with GalNAc (yellow) were identified using PDBePISA and are shown as sticks (green/cyan). Residues that have also been implicated as significant for APN binding are coloured cyan. Hydrogen bonds are formed between Cry1Ac Gln509, Arg511, Arg590 and GalNAc.

**Figure 6.**
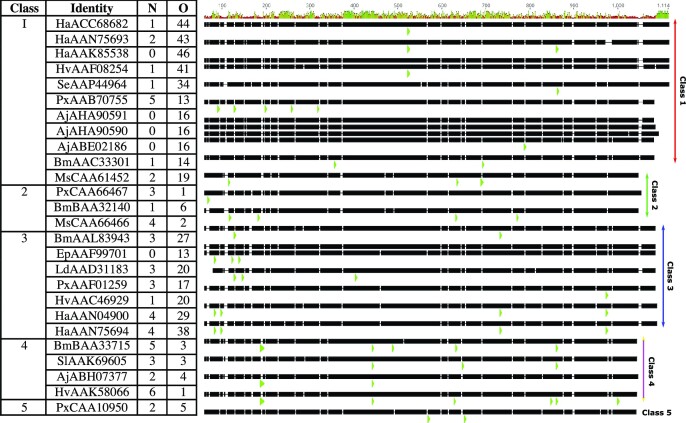
Predicted N- and O-linked glycosylation sites of lepidopteran APNs. Representative lepidopteran APN sequences that have been reported as putative Cry toxin receptors were taken from Fonseca et al. (2015), and predictions for the number of putative N-glycosylation sites (N) and O-glycosylation sites identified using the NetNGlyc 1.0 and NetOGlyc 4.0 servers (DTU Bioinformatics), respectively (table). Species abbreviations: Ha, *Helicoverpa armigera*; Hv *Helicoverpa punctigera*; Se, *Spodoptera exigua*; Px, *Plutella xylostella*; Bm, *Bombyx mori*; Ms, *Manduca sexta*; Ld, *Lymantria dispar*; Sl, *Spodoptera litura*; Aj, *Achaea janata*; and Ep, *Epiphyas postvittana*. Genbank accession numbers are shown for each protein. To visualize the placement of N-glycosylation sites, multiple sequence alignment was produced using Geneious. Mean pairwise identity is shown at the top of the alignment—green indicates 100% identity, yellow indicates between 30% and 100% identity, and red indicates below 30% identity. Green arrows show the location of predicted N-glycosylation sites (larger arrows are due to gaps in the sequence alignment).

The crystal structures of Cry1Ac and Cry1Ac in complex with GalNAc have been published (Fig. 5) (Derbyshire et al. 2001) and although this has provided evidence of D-III involvement in GalNAc binding, exactly where the GalNAc receptor ligand is located on APN is unknown. Sequence analysis of class 1 Cry1Ac-binding *M. sexta* APN isoforms showed the presence of 4–7 potential N-linked glycosylation consensus sites and 13 putative O-glycosylation sites (Knight et al. 1995, 2004, Stephens et al. 2004). A total of 10 of the putative O-linked sites are predicted in a Thr/Pro rich region of the C-terminus, thought to form a ‘stalk’ that raises the active site above the membrane. Lectin recognition of these *M. sexta* Apn1-linked glycans indicated the presence of fucosylated and high mannose N-glycans (ConA, AAA, GNA, and UEA1 lectin binding), and O-linked glycans (SBA lectin binding) (Denolf et al. 1997, Knight et al. 2004). As presented in Fig. 6, the presence of N- and O-linked glycosylation sites can be predicted by sequence analysis. Comparing the sequences of Cry-binding lepidopteran midgut APNs we see the number of N-glycosylation sites does not vary dramatically between classes (0–6 sites per protein), and the positioning of these sites is somewhat similar—especially between members of the same class. The number of O-linked sites does differ dramatically between sequences (1–46 sites), with classes 1 and 3 sequences containing substantially more consensus sites (13–46) than classes 2, 4, and 5 (1–6). Previous analysis of lepidopteran APN sequences using an earlier version of O-glycosylation site prediction software (NetOGlyc v3.1, opposed to v4.0) predicted no consensus sites for class 2 receptors (Pigott and Ellar 2007).

Individual species of N-linked glycoconjugates on the 120-kDa *M. sexta* Apn1 have been identified through MALDI-TOF/TOF tandem mass spectrophotometry coupled with lectin binding and exoglycosidase digestion. These included the common insect paucimannose structure (Man_3_GlcNAc_2_) linked to Asn609, and highly fucosylated structures at the other three consensus sites (Asn295, Asn623, and Asn752). These glycans were shown to display up to a trifucosylated core and fucosylated antennae structures (Fuc_1–3_GlcNAc). This predominance of Fucα1,3GalNAc-Asn is further indicated by the resistance of APN to PNGase F—an enzyme that cleaves all asparagine-linked oligosaccharides unless the core contains an α1,3 fucose (Stephens et al. 2004). It is unlikely that these high-fucose glycans are responsible for Cry1Ac binding as they lack terminal GalNAc residues, suggesting it is the C-terminal O-site glycans that might determine Cry1Ac binding. Supporting this hypothesis, Cry1Ac is not reported to bind to any class 2 lepidopteran APNs—a class which has significantly fewer predicted O-linked glycosylation sites and no C-terminal stalk region (Fig. 6) (Pigott and Ellar 2007). Although O-glycosylation sites have been hypothesized to be critical for Cry1Ac activity, there is evidence of Cry1Ac binding and activity in APN classes with comparatively low numbers of O-glycosylation consensus sites. Cry1Ac can bind to a class 4, 110 kDa APN present in *H. virescens* BBMV, that does not contain a C-terminal stalk and is not recognized by SBA (Banks et al. 2001). Furthermore, a class 5 APN isolated from *Athetis lepigone* (AlAPN5) has recently been identified as a putative functional receptor mediating Cry1Ac toxicity (Wang et al. 2017b). This may indicate that the increased O-glycosylation sites seen in classes 1 and 3 are not responsible for Cry1Ac toxicity, although, to the best of our knowledge, it is unknown whether Cry1Ac binding to AlAPN5 is GalNAc-dependent. Further investigations are required to determine if glycosylation is required for Cry1Ac binding to classes 2 and 5 APNs, or if these receptors work via a GalNAc-independent route.

An array of studies has shown lepidopteran APNs, of all classes, are attached to the membrane via glycosyl-phosphatidylinositol (GPI) anchors (Gill et al. 1995, Knight et al. 1995, Valaitis et al. 1995, Denolf et al. 1997, Hua et al. 1998). GPI-anchors contain carbohydrate-rich structures, often including core-linked GalNAc present at the membrane surface, leading to speculation that this may be a Cry1A binding epitope. However, removal of the GPI-anchor glycan moiety using phospholipase C (PLC) does not appear to alter binding activity (Masson et al. 1995), although it does drastically reduce Cry1Ac pore-forming activity—as expected by loss of membrane association (Lorence et al. 1997). GPI-anchored proteins, including APN, are preferentially clustered in glycolipid-enriched microdomains—specialized detergent-resistant membrane microdomains present in both mammals and insects that are enriched in cholesterol and GSLs. Chemical analysis of the 115-kDa *M. sexta* APN-associated lipid aggregate showed a predominance of neutral lipids, mainly diacylglycerol and free fatty acids (Sangadala et al. 2001). The presence of neutral lipids is interesting given the aforementioned studies indicating a reduction in neutral GSLs in resistant populations of *P. xylostella* and *M. sexta* (Kumaraswami et al. 2001, Higuchi et al. 2007). Reconstitution of the 115-kDa *M. sexta* APN into liposomes showed increased Cry1Ac binding when the lipid aggregate was present, as well as preferential binding of Cry1Ac to lipid microdomains (Sangadala et al. 2001). This concentration of APNs to lipid microdomains is hypothesized to facilitate toxin oligomerization through the high density of binding epitopes. Oligomerization of Cry1Ac and Cry1Ab is shown to facilitate membrane insertion and pore formation via significantly increasing the binding affinity to APN (~100-fold over the monomeric form) (Pardo-Lopez et al. 2006). Nevertheless, these lipid domains could also be required for protection from gut proteases or APN structural stabilization. Furthermore, lipid rafts appear to be required for the pore-forming actions of GalNAc-insensitive Cry1Ab (Zhuang et al. 2002), indicating they are not simply just enhancing toxicity via increasing GalNAc receptor concentration.

The exact role of APN and glycoconjugates in facilitating Cry1A toxicity is yet to be fully understood, with several studies indicating APN binding alone is not always enough to induce toxicity. For example, Banks *et al*. showed *Drosophila* S2 cells transfected with a novel 110 kDa APN from *H. virescens* conferred binding but did not induce pore formation (Banks et al. 2003). Furthermore, removing APN binding does not necessarily eliminate all binding, with Lee et al. (1996), showing APN competes for Cry1Ac binding with *Lymantria dispar* BBMV—but does not eliminate it. However, a significant number of reports indicate APN is critical for pore-formation (Sangadala et al. 1994, Schwartz et al. 1997, Gill and Ellar 2002). For example, expression of the 120-kDa *M. sexta* APN in the mesodermal and midgut tissue of *Drosophila* is capable of rendering normally insensitive larvae susceptible to Cry1Ac (Gill and Ellar 2002). Furthermore, several studies show that artificial APN suppression confers Cry1A resistance in several Lepidoptera (Qiu et al. 2017b, Sun et al. 2020). Divergent outcomes between these experiments are partially explained by the use of different experimental systems conferring differing posttranslational modifications—especially when we know the gut tissue is the *in vivo* target. Carroll et al. (1997), first proposed a GalNAc sensitive and a GalNAc insensitive Cry1Ac binding mechanism within the same gut, by exploring the difference in Cry1Ac binding to BBMV isolated from either the anterior (A-BBMV) or posterior (P-BBMV) midgut of a target insect, *M. sexta*. Cry1Ac binding to P-BBMV induced a faster rate of toxicity, compared to A-BBMV, but was substantially reduced by the presence of GalNAc, whilst A-BBMV binding was not. Furthermore, Cry1Ac binding to APN was concentrated in P-BBMV suggesting the GalNAc-sensitive mechanism involves APN, whilst the GalNAc-insensitive binding does not. Indeed, later studies by Banks et al. (2001) supported this idea showing that Cry1Ac recognized a distinct 110 kDa APN in *H. virescens*, where binding was not inhibited by GalNAc and the receptor itself did not bind SBA. Furthermore, a mutant Cry1Ac with an altered GalNAc binding pocket demonstrated enhanced binding to the 110-kDa APN variant, even though binding was abolished to the GalNAc-mediated 120 and 170 kDa *H. virescens* APN variant.

As briefly discussed above, a model of how APN confers Cry1Ac toxicity is through a bivalent sequential binding mechanism, with an initial low-affinity, rapidly reversed interaction (GalNAc-sensitive) followed by a slower high-affinity irreversible interaction (GalNAc insensitive) (Cooper et al. 1998, Jenkins et al. 2000). Combined mutational, binding and toxicity studies have enabled the identification of residues important for Cry1Ac binding to APN and GalNAc (Fig. 5 and Table 2). Broadly, D-I is associated with insertion of the pore into the membrane, and APN binding epitopes are primarily localized to Cry1A D-II and D-III (Rajamohan et al. 1996a, b, c, Vachon et al. 2004, Liu and Dean 2006). Domain II has been shown to influence membrane insertion, via a high affinity interaction with APN, whereas D-III is hypothesized to be involved in host specificity and the initial low-affinity receptor recognition (Wu and Dean 1996, de Maagd et al. 1999a, b)—such as the GalNAc-dependent binding mechanism of Cry1Ac (Burton et al. 1999, de Maagd et al. 1999b, Jenkins et al. 2000). Indeed, sequence analysis has shown D-III to be markedly divergent in Cry1Ac compared to other related—non-GalNAc binding—3D-Cry proteins (Bravo et al. 1997, Thompson et al. 1995).

**Table 2.** Summary of mutagenesis studies implicating a role for Cry1Ac residues in APN binding and/or Cry1Ac toxicity against *L. dispar, M. sexta*, and *H. virescens*.

Mutation	Domain	*L. dispar*	*M. sexta*	*H. virescens*
N135Q	I	–	Abolished toxicity, reduced binding to APN (phase 2), slower rate of membrane permeabilization (Cooper et al. 1998)	–
R281A	II	Reduced toxicity, reduced binding to APN (phase 2) (Jenkins et al. 2000)	–	–
R289A	II	Reduced toxicity, reduced binding to APN (phase 2) (Jenkins et al. 2000)	–	–
R368A, R369A	II	Reduced toxicity, almost abolished binding to APN (phase 2) (Jenkins et al. 2000)	–	–
R368E, R369E	II	Reduced toxicity, reduced binding to APN (phase 2) (Jenkins et al. 2000)	–	–
I375A	II	Slightly increased toxicity * (Jenkins et al. 2000)		
N377A	II	Reduced toxicity, reduced binding to APN (phase 2)	–	–
S438A–S443A	II	Reduced toxicity, reduced binding to APN (phase 2) (Jenkins et al. 2000)	–	–
S503G**	III	–	Reduced toxicity (Aronson et al. 1995)	Reduced toxicity (Aronson et al. 1995)
S503I**	III	–	Reduced toxicity, reduced binding to BBMVs (Aronson et al. 1995)	Reduced toxicity, reduced binding to BBMVs (Aronson et al. 1995)
S504R**	III	–	Reduced toxicity (Aronson et al. 1995)	Reduced toxicity
S504I**	III	–	Reduced toxicity, reduced binding to BBMVs (Aronson et al. 1995)	Reduced toxicity, reduced binding to BBMVs (Aronson et al. 1995)
N506D	III	–	Retained toxicity, reduced binding to APN, slower rate of membrane permeabilization (Burton et al. 1999)	–
N506D, Q509E	III	–	Retained toxicity, reduced binding to APN, slower rate of membrane permeabilization (Burton et al. 1999)***	–
N506D, Q509E, Y513A	III	–	Retained toxicity, abolished binding to APN, slower rate of membrane permeabilization, binding no longer inhibited by GalNAc (Burton et al. 1999)***	–
Q509E	III	–	Retained toxicity, reduced binding to APN, slower rate of membrane permeabilization (Burton et al. 1999)	–
Q509A	III	Reduced toxicity, reduced binding to BBMVs (Jenkins et al. 2000, Lee et al. 1999)	Reduced toxicity, reduced binding to BBMVs (Lee et al. 1999)	Retained toxicity, reduced binding to BBMVs (Lee et al. 1999)
Q509S	III	–	Retained toxicity, reduced binding to APN, slower rate of membrane permeabilization (Burton et al. 1999)	–
Q509A-R511A	III	Reduced toxicity, reduced binding to BBMVs, reduced binding to APN (phase 1) (Jenkins et al. 2000, Lee et al. 1999)	Reduced toxicity, reduced binding to BBMVs (Lee et al. 1999)	Reduced toxicity, reduced binding to BBMVs (Lee et al. 1999)
R511A	III	Reduced toxicity, reduced binding to BBMVs, reduced binding to APN (phase 1) (Jenkins et al. 2000, Lee et al. 1999)	Reduced toxicity, reduced binding to BBMVs (Lee et al. 1999)	Reduced toxicity, reduced binding to BBMVs (Lee et al. 1999)
Y513A	III	Reduced toxicity, reduced binding to BBMVs, reduced binding to APN (phase 1) (Jenkins et al. 2000, Lee et al. 1999)	Reduced toxicity, reduced binding to BBMVs and APN, slower rate of membrane permeabilization (Burton et al. 1999, Lee et al. 1999)	Retained toxicity, reduced binding to BBMVs (Lee et al. 1999)
W545A	III	Reduced toxicity, abolished binding to APN (phase 1), abolished GalNAc recognition (Jenkins et al. 2000)	Retained toxicity, reduced binding to APN (Pardo-Lopez et al. 2006)	–

*95% confidence intervals overlapped with wildtype Cry1Ac.

**When these residues are mutated to an alanine, no differences in toxicity or binding are observed in *L. dispar, M. sexta*, or *H. virescens* (Lee et al. 1999).

***Further decreased rate of membrane permeabilization that the previous mutation presented in the table.

The first phase of APN recognition is hypothesized to be through fast, low affinity D-III binding. This is supported by Lee et al. (1999), who generated a series of alanine substitution mutations in the region of D-III unique to Cry1Ac (503–525 aa) and demonstrated that binding affinity was significantly reduced, and to a relatively greater degree than toxicity (Fig. 5B). Whilst some of these mutant residues are in direct contact with GalNAc (Q509, R511, and Y513,), others are not (S503, S504, N506, N510, and W545)—but with the exception of W545 are in close proximity to the binding pocket. It was not investigated whether any of these mutations affect GalNAc binding, making it difficult to interpret whether reduced mutant binding to BBMVs was through a loss of GalNAc binding. The authors conclude that if D-III is predominantly involved in initial low-affinity APN binding, then this will only compromise second phase high-affinity binding when it is reduced by at least 5-fold. Burton et al. (1999) also reported substitution mutations in the unique region of Cry1Ac D-III (N506D, Q509E, and Y513A—the latter two having direct contact with GalNAc in the crystal structure of the complex) resulted in reduced binding and slower pore formation, with the triple mutation no longer inhibitable by GalNAc—yet no significant differences in toxicity were observed. Further supporting that D-III binding is required for sequential D-II binding, the mutation of a tryptophan residue (W545A) in D-III (Fig. 5B) can completely abolish sequential binding of D-II to the *L. dispar* APN and recognition of GalNAc—of particular note given W545 is not part of the GalNAc binding pocket (Jenkins et al. 2000). Interestingly, all Cry1Ac tryptophan residues are conserved in the closely related Cry1Ab, except the D-III W545 residue (Rausell et al. 2004). The complete loss of APN binding in *L. dispar*, via the Cry1Ac W545A mutation, only caused a 50-fold decrease in activity, whereas the same W545A mutation in *M. sexta* larvae did not abolish binding to APN, with little to no loss in toxicity (Pardo-Lopez et al. 2006). The work in *M. sexta* also demonstrated that GalNAc binding to the Cry1Ac oligomer increases the exposure of W545 to solvent, through a subtle conformational change in the GalNAc binding pocket region of D-III. In *M. sexta*, this conformational change is hypothesized to be responsible for the marked increase in binding affinity of the Cry1Ac oligomer to APN. Collectively, these data indicate that D-III functions to bind both GalNAc and APN in a low affinity manner, which can affect second-phase APN binding, yet there are apparent species-specific differences which determine Cry1Ac interaction with APN and toxicity, and an indication that Cry1Ac can retain toxicity even when binding to APN and GalNAc is abolished—leaving the binding open to further investigation.

Domains II and III are not specifically linked to glycan interactions, yet a common theme is apparent between mutational studies in all three domains; the binding to APN and subsequent toxicity are not necessarily correlated. This could be explained by the presence of alternative *in vivo* Cry1Ac receptors—such as cadherin-like receptors or ABC transporters—that function independently of APN and could be potentially compensating for the lack of APN binding/activity. The exact model used may change the distribution/concentration of APN and any potential alternative receptors. Furthermore, the exact experimental setup may play a significant role. If APN binding to D-III is the rate limiting step to binding to D-II, and D-II binding and membrane permeabilization is not abolished but slowed, it may be possible to exert toxicity over a longer time course. A better understanding of the key residues in Cry1Ac required for binding to receptors, and the role of GalNAc in this binding, might enable improved engineering of both insect specificity and toxicity, as well as providing a valuable tool for identifying potential resistance-driving mutations.

### Cry1A binding to cadherin-like receptors

Vadlamudi et al. (1995), purified and characterized the first cadherin-like receptor from *M. sexta* larvae, a 210-kDa protein termed BT-R_1_. Sequence analysis showed a 30%–60% similarity to the cadherin superfamily of proteins—a large family of transmembrane glycoproteins characterized by repeated calcium-binding domains. Since the discovery of BT-R_1_, receptors with a highly similar domain organization have been identified in an array of other lepidopteran species including *B. mori* (BtR175), *H. virescens* (HevCaLP), *O. nubilalis, L. dispar, P. xylostella* (PxCad), *C. suppressalis* (CsCad), and *H. armigera* (HaCad) (Nagamatsu et al. 1999, Gahan et al. 2001, Morin et al. 2003, Flannagan et al. 2005, Wang et al. 2005a, Xu et al. 2005). Lepidopteran cadherin receptors are usually anchored to the apical membrane of the midgut epithelium via a single transmembrane domain and, unlike GPI-anchored receptors (such as APNs or ALPs), are not preferentially localized to glycolipid-enriched lipid microdomains (Zhuang et al. 2002, Midboe et al. 2003). Interestingly, Cry1Ab treatment of *M. sexta* microvilli membranes was shown to induce Bt-R_1_ localization to lipid microdomains—although this is likely due to Bt-R_1_ remaining attached after toxin oligomerization and not due to a requirement for (glyco)lipid-facilitated binding (Bravo et al. 2004).

There are significant data to show cadherin-like receptors function in determining Cry1A specificity and toxicity in lepidopteran larvae (Pigott and Ellar 2007) and lepidopteran and *Drosophila*-derived cell lines (Keeton and Bulla 1997, Hua et al. 2004, Zhang et al. 2005). Furthermore, expression of BT-R_1_ and BtR175 in mammalian-derived cell lines can induce Cry1Ac toxicity (Dorsch et al. 2002, Tsuda et al. 2003), suggesting cadherin-like receptors alone may be enough to permit cytocidal action and no other ‘insect-specific’ features are required for action. The success of inducing Cry1A toxicity in cell lines through cadherin-like receptor expression alone may be due to the redundancy of glycosylation in specifying binding. Unlike APN, there are no reports of sugars acting as binding competitors with Cry1Ac to cadherin-like receptors. Further indication that glycosylation is not required comes from a study showing that the shortest fragment of Bt-R_1_ that binds Cry1A toxins is a nonglycosylated 169 aa ectodomain fragment, i.e. also capable of inhibiting toxicity (Dorsch et al. 2002). To the best of our knowledge, the current literature does not report glycosylation to play a significant role in cadherin-like receptor binding, although N- and O-linked glycosylation sites are present on all identified lepidopteran cadherin-like receptors (Shao et al. 2018).

### Cry1A binding to ALP receptors

Selection of a Cry1Ac resistant strain of *H. virescens* allowed for comparison of midgut epithelium proteins between susceptible (YHD2) and resistant (YHD2-B) larvae (Jurat-Fuentes et al. 2002, Jurat-Fuentes and Adang 2004). After observing reduced Cry1Ac binding to YHD2-B BBMVs, based on the rationale that GalNAc forms part of the Cry1Ac receptor, the authors investigated levels of SBA binding to BBMVs and indeed observed reduced SBA binding to YHD2-B resistant larvae—initially indicative of altered glycosylation (Jurat-Fuentes et al. 2002). Further characterization of YHD2-B BBMVs identified a 68-kDa glycoprotein as a GPI-anchored alkaline phosphatase—HvALP. Digestion of BBMV proteins with PNG-F to release N-terminal oligosaccharides, eliminated SBA binding to HvALP, confirming the presence of N-linked oligosaccharides with terminal GalNAc residues. Addition of Cry1Ac abolished SBA binding to HvALP, indicating competitive binding of both proteins for the same N-linked GalNAc residues on HvALP. Correlating with reduced Cry1Ac binding, Cry1Ac-resistant BBMVs also demonstrated a reduction in expression and a 3-fold decrease in activity of HvALP—suggesting the resistance was not due to altered glycosylation or recognition of GalNAc, but instead due to a reduction in HvALP protein expression—although the authors did not perform oligosaccharide analysis, resistance through altered glycosyl interactions cannot be completely ruled out (Jurat-Fuentes and Adang 2004). In a parallel with the work described above, Ning et al. 2010 described two ALPs cloned from *H. armigera* (HaALPs) that specifically bind Cry1Ac via N-linked GalNAc. Whether GalNAc binding on ALP is required for Cry1Ac toxicity is still open for debate—indeed GalNAc addition to *H. armigera* BBMVs inhibits permiabilization (Rodrigo-Simon et al. 2008), however, whether this is directly through ALP and the relevance to *in vivo* activity is yet to be determined.

## Cry5B and Cry14A

Cry5B is the best-characterized of the Cry5 subfamily of six phylogenetically related proteins (Cry5Aa, Cry5B, Cry12A, Cry13A, Cry14A, and Cry21A) that may demonstrate nematocidal and/or insecticidal activity (Wei et al. 2003). Consistent with the mode of 3D-Cry protein insecticidal toxin actions, susceptible nematodes fed with nematocidal Bt strains experience dose-dependent lethality associated with reduced feeding activity, inhibited development and intestinal damage. To date, both Cry5B and Cry14A nematocidal activity is shown to be dependent, at least in part, on glycolipids (Marroquin et al. 2000, Griffitts et al. 2001, 2003, 2005).

Using forward genetics in *C. elegans*, Marroquin et al. (2000) identified five *bre* genes (for *Bacillus*-toxin resistant), four of which confer high levels of resistance to Cry5B induced toxicity and one (*bre-1*) that confers a significantly lower level. In all resistant mutants, Cry5B toxin remained in the intestine and was not internalized into the gut cells indicating resistance via reduced ‘receptor’ binding. The first *bre* gene to be characterized was *Bre-5*, found to encode a β1,3-galactosyltransferase with strong sequence similarity to the *Drosophila brn* gene (required for glycolipid synthesis; see Fig. 4) (Griffitts et al. 2001). Successively, *bre-2, bre-3*, and *bre-4* were characterized as encoding further glycolipid synthetic proteins; *bre-4* as a UDP–GalNAc:GlcNac β1–4-*N*-acetlygalactosaminyltransferase, *bre-2* encodes a β1,3 glycosyltransferase, and *bre-3* a putative glycosyltransferase homologous to *Drosophila egh* (see Fig. 4) (Griffitts et al. 2003, 2005). Functional homology of *bre* genes to the *egh-brn* invertebrate-specific lipid glycosylation pathway was shown via TLC lipid analysis, demonstrating that *bre* mutants express no (*bre-3, bre-4*, and *bre-5*), or significantly reduced (*bre-2*) complex GSLs, yet have no change in N- or O-linked proteoglycan profiles. Specific binding of Cry5B to these *bre*-dependent complex GSLs alongside genetic epistasis-based experiments supported the proposal that *bre*-genes act consecutively (*bre-3, bre-4, bre-5*, and *bre-2*) to synthesize a functional lipid-linked oligosaccharide receptor with terminal galactose residues (Griffitts et al. 2005). In further support of GSLs as principal determinants for Cry toxicity, the *C. elegans* LEC-8 galectin (a ß-galactoside-binding protein) can compete with Cry5B for carbohydrate binding. Cry5B binding to *C. elegans* glycolipid-coated TLC plates was inhibited through the addition of recombinant LEC-8, and *C. elegans* LEC-8 deficient mutants were more susceptible to Cry5B, in comparison to wild type worms (Ideo et al. 2009). *Bre* mutants also demonstrated a moderate resistance to Cry14A, a toxin with 34% sequence identity to Cry5B in their protoxin forms and ∼30% identity in the activated form. This relatively low level of amino acid identity suggests that other distantly related toxins may induce *bre*-mediated toxicity. However, the reduced resistance, compared to Cry5B, signifies that other Cry14A receptor(s) may compensate for the loss of the *bre*-mediated glycolipid (Griffitts et al. 2001, 2003).

Although identified in the same forward genetics screen as *bre 2–5, bre-1* mutants demonstrate substantially less Cry5B resistance Marroquin et al. 2000, Barrows et al. 2007). *Bre-1* has since been identified as a GDP-mannose 4,6 dehydratase (GMD), an enzyme involved in a fucose salvage pathway. Unlike the *bre2–5* genes, it does not function in a glycolipid-specific manner, with *bre-1* defective mutants showing strikingly reduced levels of fucosylated N and O-linked proteoglycans as well as fucosylated glycolipids (Barrows et al. 2007). This partial Cry5B resistance indicates that fucose is less critical for eliciting Cry5B binding than terminal galactose residues—as shown by competitive binding studies.

Interestingly, no obvious change in phenotype or lethality were observed in the *bre*-mutant *C. elegans*, apart from a small reduction in brood size in *bre-1* and *bre-3* worms (Barrows et al. 2007). The nematode is apparently capable of surviving with reduced levels of GSLs and dramatically reduced fucose, which is perhaps surprising given the commonality of fucose in nematode glycans and the prevalence of detrimental phenotypes in mammalian GMD knockouts (Keeley et al. 2019, Sturla et al. 2001). This has implications for Cry resistance in nematodes, since they can tolerate changes in glycosylation while in *Drosophila*, the equivalent *brn* and *egh* mutants are lethal/sterile, suggesting a significantly lower tolerance to reduced *bre*-mediated glycosylation and an essential role for GSLs in insects. This contrast in phenotypes could suggest that insects, in contrast to nematodes, would be less able to achieve to Cry resistance via GSL alteration.

## Cry2

Like the Cry1 class of Bt proteins, Cry2 proteins are largely specific towards lepidopteran insects (Hernandez-Rodriguez et al. 2008), with some Cry2A variants also exhibiting toxicity against mosquito species, including *Ae. aegypti, Culex quinquefasciatus, Anopheles stephensi*, and *An. gambiae* (Moar et al. 1994, Sims et al. 1997, Misra et al. 2002, McNeil and Dean 2011, Ricoldi et al. 2018, Goje et al. 2020, Valtierra-de-Luis et al. 2020). Whilst Cry2 is not reported to bind any APNs, ALPs, or CADs, functional Cry2A ABC receptor binding proteins have been identified—ABCC1 and ABCA2 from *H. armigera* (HaABCC1 and HaABCA2) and ABCA2 from *B. mori* (BmABCA2) (Wang et al. 2017a, Chen et al. 2018, Li et al. 2020), *P. gossypiella* (PgABCA2) (Fabrick et al. 2021), and *Helicoverpa zea* (HzABCA2) (Fabrick et al. 2022). The ABC transporter superfamily of proteins are responsible for the ATP-powered translocation of a diverse assortment of substrates across membranes. In common with shared physiological mechanisms observed with mammalian ABC transporters, insect ABC transporters have been functionally linked to lipid transport, and the transport of xenobiotics and their metabolites (Rees et al. 2009, Broehan et al. 2013).

Sequence analysis of HaABCC1 showed the presence of 14 potential N-glycosylation sites and 16 potential O-glycosylation sites throughout the entire protein (Chen et al. 2018). In HaABCA2, sequence analysis identified six potential N-glycosylation sites within the extracellular (EC) domain loops of transmembrane domain (TMD)-1 and TMD-2 (Tay et al. 2015). One of these putative N-glycosylation sites is located within a 5-bp deletion mutation shown to confer Cry2Ab resistance in *H. armigera*. This deletion mutation introduces a stop codon within HaABCA2 TMD-2, leading to a protein truncation. Although the use of these these putative glycosylation sites is yet to be confirmed, it has been hypothesized that binding of Cry2A toxins to the glycosylated EC domain loops of ABCA2 may form the basis of toxin oligomerization and sequential pore formation (Tay et al. 2015). Given that ABC transporters have been shown to exist as multiprotein complexes in the membrane, it may also be the case that other ABC-associated proteins are involved in Cry2A binding and pore-formation (Kaminski et al. 2006). Other reported Cry2 receptors include the Se-V-ATPase subunit B from *S. exigua*, also predicted to contain several putative glycosylation sites (Qiu et al. 2017a).

Cry2Ab has also been shown to interact with lipophorin glycolipids (Ma et al. 2012b). As discussed previously, Ma et al. (2012b) demonstrated that Cry1Ac addition to *G. mellonella* lipid particles induced aggregation, and sequesters Cry1Ac to the gut lumen, possibly increasing Cry toxin tolerance. Cry2Ab was also shown to aggregate following lipid particle interaction and, hence, the authors suggest a similar mechanism of toxin tolerance.

## Cry3

The Cry3 class is the best-characterized of the coleopteran-specific proteins, with a domain architecture consistent with other 3D-Cry proteins. The lectin-like D-III of Cry3Aa was found to exhibit strong resemblance to the N-terminal cellulose binding domain (CBD_N1_) of the bacterial *Cellulomonas fimi* 1,4-β-glucanase C (CenC) (Johnson et al. 1996, Burton et al. 1999). The CBD_N1_ domain of CenC has been shown to interact with cellulose, as well as cell oligosaccharides and β-1,4-linked oligomers of glucose (Tomme et al. 1996)—with binding thought to occur via β-strands within a five-stranded cleft which constitutes the CBD_N1_ (Johnson et al. 1996, Kormos et al. 2000). The structural correlation between Cry3 D-III and the CBD_N1_ of CenC may suggest a role for sugar moieties in Cry3 receptor binding.

Several studies have implicated CADs, ALPs, APNs, and ABCs as Cry3 binding proteins and/or functional receptors—although less is known regarding the relevance of glycosylation. In *Tenebrio molitor*, Cry3Aa has been shown to bind to a GPI-anchored ALP, which is preferentially expressed in the BBMV of early instar larvae (Zuniga-Navarrete et al. 2013). In *D. virgifera virgifera* and *Chrysomela tremula*, ABCB1 has been identified as a functional receptor for Cry3A (Niu et al. 2020). Functional validation of the *D. virgifera virgifera* Cry3A receptor (DvABCB1) was achieved through activated Cry3A addition to Sf9 or HEK293 cells, both expressing DvABCB1. As the gut of *D. virgifera vigifera* is mildly acidic, this again indicates the pH of cell studies does not need to replicate the gut environment for toxicity to occur, in the presence of activated toxin. Sequence analysis of CtABCB1 predicts two putative glycosylation sites on the EC loops of the transmembrane domains (Pauchet et al. 2016). Although the functional relevance of these sites is unknown, this is the first study to suggest that glycosylation may be important for ABC receptors.

## Cry30Ca2

Cry30Ca2 is produced by the Bt serovar. *jegathesan*, a mosquitocidal subspecies that shows toxicity against *Ae. aegypti, An. stephensi, Culex pipiens*, and *C. quinquefasciatus* (Delecluse et al. 1995, Kawalek et al. 1995). Bioassays of the isolated Cry30Ca2 toxin indicate that this individual protein is not toxic against *C. quinquefasciatus* and, hence, additional studies are required to test its toxicity to other mosquitoes (Sun et al. 2013).

Using homology modelling, based upon Cry4Ba, Zhao et al. (2012) produced a 3D model of the Cry30Ca2 structure consistent with that of other 3D-Cry proteins. Dissimilar from the interaction of Cry1Ac with GalNAc, which occurs via Cry1Ac D-III, docking studies investigating the interaction of Cry30Ca2 with GalNAc highlighted a distinct, putative binding site within the apical loops of the Cry30Ca2 lectin-like D-II (residues I321 in loop 1, Q342, T343, Q345 in loop 2, and Y393 in loop 3, which form seven hydrogen bonds with GalNAc) (Zhao et al. 2012). Various studies have implicated the loop regions of Cry protein D-II in receptor binding, including Cry3Aa, which is shown to bind TmCad1 via D-II loop 1 (Zuniga-Navarrete et al. 2015). Given the results of molecular docking studies and these structural similarities, a role for GalNAc containing carbohydrate moieties in Cry30Ca2 mosquitocidal activity has been suggested (Zhao et al. 2012). However, the effect of GalNAc on the activity of Cry30Ca2 is yet to be investigated.

## Cry11a

Bt serovar. *israelensis* (Bti) strains are highly toxic to a number of mosquito species and, as such, are used for the control of their populations in the field (Mittal et al. 2003). One such Bti toxin is Cry11Aa, which displays toxicity against *Aedes* and *Culex* larvae and, to a lesser extent, *Anopheles* larvae (Otieno-Ayayo et al. 2008). Cry11Aa has been identified to bind receptors in mosquito larvae that are in the same classes as Cry toxins that act against Lepidoptera, including APNs, ALPs, and Cadherins.

Cry11Aa binding to an ALP is suggested to play a role in mediating toxicity in *Aedes* larvae (Fernandez et al. 2006). Interestingly, the interaction between Cry11Aa and *Ae. aegypti* ALP1 (AaeALP1) was shown to be modulated by other proteins—namely C-type lectins and galectins—which both interfere with toxicity (Batool et al. 2018, Zhang et al. 2018). C-type lectins are a superfamily of proteins that have mannose and galactose type carbohydrate binding capabilities through conserved residues (Brown et al. 2018). Galectins are a family of proteins that typically bind to β-galactoside carbohydrates (Modenutti et al. 2019), although comparatively little is known about their carbohydrate binding properties and function in invertebrates compared to vertebrates (Yang et al. 2011, Zhang et al. 2018). The *Ae. aegypti* C-type lectin-20 (CTL-20) can bind to both *Aedes* BBMVs and recombinant AaeALP1, in addition to binding to Cry11Aa itself. Further to this, CTL-20 has been shown to compete with Cry11Aa for binding to AaeALP1 suggesting that they bind AaeALP1 in the same region (Batool et al. 2018). Similarly, galectin-14 has been shown to compete with Cry11Aa for binding to AaeALP1 and *Aedes* BBMVs, with modelled molecular docking indicating that Cry11Aa and Galectin-14 bind to ALP1 on two different, but overlapping, interfaces (Zhang et al. 2018). Additionally, other galectins such as galectin-6 have also been shown to interfere with Cry11Aa toxicity (Hu et al. 2020). There is some evidence that galectin-6 binds to molecules containing galactose-β1,4-fucose (Takeuchi et al. 2008, Maduzia et al. 2011), therefore, it is possible that Cry11Aa may be able to bind similar glycan moieties. These results draw comparison with the Cry5B data discussed previously, where LEC-8 competes for carbohydrate binding and suggests a role for glycan moieties in the interactions between Cry11Aa and ALP1. However, to the best of our knowledge, there are no studies which have directly investigated the involvement of glycan residues in this binding.

Perhaps the most extensive work looking into the role of glycoconjugates in Cry11Aa receptor binding has come from Chen et al. (2009a), investigating the interactions between Cry11Aa and AaeAPN1. This study identified AaeAPN1 as a Cry11Aa binding partner through pulldown assays utilizing biotinylated toxin performed on solubilized *Ae. aegypti* BBMV. The AaeAPN1 was cloned and expressed in both *E. coli* and Sf21 cells—significant in the context of investigating the role of glycoconjugates as *E. coli* do not naturally N- and O- glycosylate proteins (Du et al. 2019). The glycosylation status of AaeAPN1 from BBMV was investigated through lectin blots (Chen et al. 2009a) and demonstrated the native form of AaeAPN1 was detectable by WGA but not SBA, indicating AaeAPN1 contains *N*-acetylglucosamine moieties but not terminal *N*-acetylgalactosamine residues. In Sf21 cells, expression of a catalytically active form of AaeAPN1 did not render cells susceptible to Cry11Aa treatment. Although Sf21 cells have the ability to N- and O-glycosylate proteins (Davis and Wood 1995), the AaeAPN1 in these cells was not detected by WGA, SBA, ligand blot, or toxin pull down assays and the band detected by anti-APN1 antibody was smaller than expected—possibly due to differences in post translational modifications (Chen et al. 2009a). The authors also hypothesized that alternative glycosylation in Sf21 cells could mask a glycan-independent binding site. Taken together these results may indicate that that glycosyl moieties are required for binding. However, Chen et al. (2009b) also demonstrated, via dot blot and competitive ELISA, that a truncated AaeAPN1 fragment expressed in *E. coli* binds to Cry11Aa, suggesting that this interaction is glycan-independent, due to the absence of N and O-glycosylation in *E. coli—*although this does not determine whether this binding is involved in mediating toxicity.

The sequence of an *Aedes* cadherin protein shown to bind to Cry11Aa has been determined and there are predicted N-glycosylation sites within the cadherin repeats, however, there has been no investigation so far into whether glycosylation is present and if it is required for this interaction (Chen et al. 2009b).

## Cry4Ba

Cry4Ba is also produced by Bti and is processed in the insect midgut to produce an active toxin of 65 kDa (Angsuthanasombat et al. 1991). Like Cry11Aa, Cry4Ba also targets *Aedes* and *Anopheles* mosquito larvae (Otieno-Ayayo et al. 2008, Ben-Dov et al. 2014,), and is shown to target the same receptor classes as other 3D-Cry toxins (APNs, ALPs, and Cadherins) (Likitvivatanavong et al. 2011, Saengwiman et al. 2011).

A cadherin Cry4Ba binding partner (AgCad1), expressed in *An. gambiae* BBMVs was predicted to be glycosylated, based upon the observed AgCad1 protein band having a slightly larger molecular weight than expected (Hua et al. 2008). The same group also demonstrated that Cry4Ba displays limited binding on dot blots to an *E. coli*-expressed truncated peptide from AgCad1 (a CR11 membrane proximal EC domain peptide), suggesting that some binding is possible in the absence of glycosylation or other *in vivo* requirements. Similarly, Cry4Ba was shown to bind to a segment of the *An. gambiae* cadherin BT-R_3_, expressed in *E. coli*, which consisted of the EC domain module 7 through to the membrane proximal EC domain (Ibrahim et al. 2013). As this cadherin fragment was expressed in *E. coli* it is unlikely to be glycosylated and provides further evidence that glycosylation of cadherins is not required for Cry4Ba binding.

Multiple studies have implicated ALPs as binding partners for Cry4Ba (Bayyareddy et al. 2009, Dechklar et al. 2011, Jimenez et al. 2012). Mutagenesis studies demonstrated Cry4Ba binding to ALP1, in part, through D-II loop II. Multiple Cry4B D-II mutants displayed reduced binding to ALP1 from BBMV and *E. coli*, and reduced toxicity to *Ae. aegypti* larvae. The results of this study suggest it is unlikely that receptor glycosylation is essential for interaction, as the mutated versions of Cry4Ba also display reduced binding to *E. coli* expressed ALP1 and *Ae. aegypti* BBMV (Jimenez et al. 2012). Further to this, Thammasittirong et al. (2011) showed that Cry4Ba binds to an *Ae. aegypti* ALP expressed in *E. coli* with high affinity, which they conclude supports the notion that Cry4Ba interactions with ALPs does not require glycosyl moieties as proteins expressed in *E. coli* are unlikely to be glycosylated. Finally, Buzdin et al. 2002 showed through ligand blots that addition of monosaccharides (mannose, glucose, galactose, galactosamine, *N*-acetylglucosamine, and *N*-acetylgalactosamine, either individually or in mixtures) did not interfere with Cry4Ba binding to ALP that was prepared from *Ae. aegypti* BBMVs, with similar results shown for Cry11Aa binding to ALP. They also demonstrated that the addition of *N*-acetylglucosamine or *N*-acetylgalactosamine failed to elute ALP from Cry4Ba- and Cry11Aa- Sepharose (Buzdin et al. 2002). APNs have also been identified as receptors for Cry4Ba (Saengwiman et al. 2011). Sf9 cells expressing two *Ae. aegypti* APN isoforms (AaeAPN2778 and AaeAPN2783) displayed increased sensitivity to Cry4Ba and the toxin was shown to bind to APNs in Sf9 cells (Aroonkesorn et al. 2015). The APNs expressed in these cells were thought not to be glycosylated, suggesting that the interaction between APNs and Cry4Ba is glycan independent.

Cry4Ba has been shown to interact directly with lipid bilayers, which is perhaps not surprising given the elucidation of GPI-anchored APN and ALP receptors. Thammasittirong et al (2019) tested full length Cry4Ba and D-III-only binding to lipid bilayers and liposomes prepared from an artificial lipid mix containing phosphatidylethanolamine, phosphatidylcholine and cholesterol (but no glycans). They focused on D-III of Cry4Ba as it is shown to bind along the apical microvilli of the larval midgut of *Ae. aegypti* (Chayaratanasin et al. 2007). Domain III of Cry4Ba displayed tight binding to immobilized liposome membranes with a K_D_ comparable to that of the full-length protein. However, unlike the full-length protein, the truncated D-III Cry4Ba fragment did not induce ion-channel formation in planar lipid bilayers or permeability of calcein dye-loaded liposomes, consistent with the role of this domain as a membrane anchor rather than having a role in pore formation (Thammasittirong et al. 2019). The binding of Cry4Ba to lipids may suggest that, like other Cry proteins, it localizes to lipid rafts—although whether glycolipid binding occurs, remains to be tested.

## Glycan binding in other bacterially produced insecticidal toxins

There is also evidence that glycan binding could play an important role in the insecticidal action of other structural classes of bacterially produced toxins, with lectin-like domains present in the Tpp family (D-I) (Colletier et al. 2016), Vegetative insecticidal protein family (Vip3, D-IV, and D-V) (Zheng et al. 2020), mosquitocidal holotoxin (Mtx1Aa1) (Treiber et al. 2008), and the membrane attack complex/perforin family (Mpf, C terminal domain) (Zaitseva et al. 2019). Sugar binding appears to play a role with several members of the Tpp family, including Tpp78, Tpp80, and the Tpp1/Tpp2 binary complex. Several sugars—including chitotriose, *N*-acetylmuramic acid, chitobiose, and *N*-acetylneuraminic acid—can reduce the mosquitocidal action of *Lysinibacillus sphaericus*-produced Tpp1/Tpp2 in *Culex* cell lines (Broadwell and Baumann et al. 1987), and arabinose and fucose can reduce Tpp1 toxicity towards *Culex* larvae (Sharma et al. 2018). Both galactose and GalNAc have recently been demonstrated to inhibit the activity of Bt-produced Tpp78 (Cao et al. 2022) and Tpp80 (Best et al. 2022) against their respective targets, rice planthoppers (*Laodelphax striatellus* and *Nilaparvata lugens*) and mosquitoes (*C. quinquefasciatus, Ae. aegypti*, and *An. gambiae*). The mosquitocidal Mtx1Aa1 contains 12-putative sugar binding domains across four ricin B-type lectin repeats, which are structurally related to Piersin—a cytotoxin, i.e. reported to bind Gb3 and Gb4 glycolipids (Matsushima-Hibiya et al. 2003). This is just a snapshot of the glycan-binding literature on other bacterial pesticidal proteins, and highlights glycan binding as an important mechanistic theme across bacterially produced pesticidal proteins.

## Conclusions

Bt 3D-Cry toxins are critical for progressing a sustainable approach to controlling pests of agriculture and vectors of human disease, with the development of field resistance threatening current effectiveness and progress. Understanding the mechanism of action is key to understanding resistance and the potential development of new 3D-Cry proteins. All known 3D-Cry proteins contain lectin-like domains, indicating a potential role for glycan-binding. For several Cry proteins, interaction with sugars, glycoproteins, glycolipids, and competition by lectins has been demonstrated in receptor binding, but a role in toxicity is not always clear. For other members of the Cry family, these studies are absent, suggesting an important gap in our knowledge that should be addressed. While for some proteins, such as Cry4B, above, binding to protein receptors appears to be glycosylation independent, the potential carbohydrate-binding properties of D-II and D-III may play a role in binding to glycolipid moieties in the target cell membrane (as shown for Cry5B). The structural differences in glycoconjugates between insects, nematodes, and mammals is a mechanistic explanation for target range, i.e. independent of the protein receptor and may explain why the transfection of genes for such receptors does not always confer susceptibility to recipient cells. This effect will be mediated by both the specificity of the carbohydrate binding domains within the Cry proteins and the natural lipid composition of the transfected cells. Understanding the exact role of glycoconjugates can be a challenge due to the difficulty in replicating the *in vivo* environment of the gut target tissue—especially with many studies suggesting a complex coordination of binding components is required to elicit the full spectrum of toxicity. Indeed, the majority of model data comes from cell lines, which are not target-tissue specific and BBMV binding studies in which the concentrations of receptors and lipid microdomains do not necessarily accurately reflect the *in vivo* environment. In addition to normal development, glycan expression can be significantly altered by environmental pressures, such as temperature, infection, and dietary changes. This should be considered in terms of the development of Bt tolerance in target species—where changes in glycan binding profiles may be an indication of resistance as observed with nematocidal Cry5B. Despite these experimental complexities, it is clear that glycan moieties might be critical for exerting insecticidal and nematocidal activity, with glycan-moieties observed as primary receptors critical for activity, and in more additive roles that can affect the spectrum/potency of activity. Despite many years of study of the Cry proteins, our understanding of their glycoconjugate interactions remains underinvestigated and in its infancy. Application of the tools of glycobiology to the study of insecticidal proteins in future will help us to resolve the importance of these interactions.
